# Spinel‐Layered Heterostructure Enables Reversible Oxygen Redox in Lithium Manganese Oxide

**DOI:** 10.1002/anie.202511054

**Published:** 2025-07-11

**Authors:** Yanfang Wang, Cheng Li, Yulin Cao, Juping Xu, Dominic Gardner, Wilgner Lima da Silva, Yongcong Huang, Fangchang Zhang, Mingzhou Li, Yingzhi Li, Wen Yin, Kaili Zhang, Phoebe K. Allan, Zhouguang Lu

**Affiliations:** ^1^ Department of Materials Science and Engineering Southern University of Science and Technology Shenzhen 518055 China; ^2^ School of Chemistry University of Birmingham Edgbaston Birmingham B15 2TT UK; ^3^ Department of Mechanical Engineering City University of Hong Kong Tat Chee Avenue Kowloon Tong Hong Kong SAR China; ^4^ Eastern Institute for Advanced Study Eastern Institute of Technology Ningbo Zhejiang China; ^5^ Chinese Academy of Sciences Institute for High Energy Physics Beijing 100049 China; ^6^ Spallation Neutron Source Science Centre Dongguan 523803 China; ^7^ The Faraday Institution Harwell Campus Didcot UK

**Keywords:** Cathode material, Lithium‐ion batteries, Oxygen redox, Spinel‐layered heterostructure

## Abstract

Lithium‐rich manganese‐based layered oxides (LRMOs) have emerged as promising cathode materials for next‐generation lithium‐ion batteries (LIBs), primarily due to their exceptional capacity originating from oxygen redox chemistry. Although Li_2_MnO_3_ (LMO) has been conventionally identified as the oxygen redox‐active component in LRMOs, this layered material shows neither bulk redox activity nor reversible anion redox behavior in the absence of other transition metals (e.g., Ni and Co). Herein, we report a structural‐engineered lithium manganese oxide with spinel‐layered heterostructures (designated as LMO‐SH), which exhibits reversible oxygen redox activities between lattice oxygen (O^2−^) and molecular oxygen (O_2_) – the first documented instance of such redox behavior in a manganese‐based material. Through combining experimental characterization and theoretical modeling, we establish that the interfacial architecture between the spinel and layered phases facilitates the Li^+^ diffusion kinetics while simultaneously activating bulk oxygen redox processes. This mechanistic understanding not only advances fundamental knowledge of redox chemistry in LMO‐based materials but also establishes new design principles for developing high‐capacity cathodes through strategic phase engineering.

## Graphical abstract

A layered lithium manganese oxide with spinel heterostructures (LMO‐SH) was prepared via a novel synthetic method. It has lithium‐rich local environments and exhibits reversible oxygen redox activities, distinct from the conventional Li_2_MnO_3_. The spinel heterostructure provides extra Li^+^ pathways to activate the bulk activity and traps oxygen molecules (O_2_) for reversible reduction.

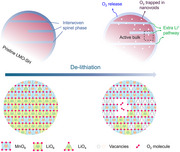

## Introduction

Driving toward a sustainable future requires better lithium‐ion batteries (LIBs) with high energy densities. Intensive efforts have been devoted to exploring extra capacities from oxygen redox activity in addition to traditional cation redox, as demonstrated in plenty of lithium‐rich cathodes.^[^ ^1^ ^]^ Among them, lithium‐rich manganese‐based layered oxides (LRMOs, Li_1+x_TM_1‐x‐y_Mn_y_O_2_, TM = Ni, Co, Al, etc.) are arguably the most favorable candidates thanks to their high‐capacity, environmental benignity, and cost‐effectiveness.^[^ ^2^, ^3^ ^]^ Although it is still under debate, LRMOs are broadly understood to be biphasic mixtures of typical LiTMO_2_ (TM = Ni, Co, Mn, Al, etc.) and lithium‐rich Li_2_MnO_3_ on the atomic/ nano scale, in which the latter is widely considered to be the anionic active component.^[^ ^4^ ^]^ LRMOs can deliver excess capacities contributed by reversible oxygen redox activities, as evidenced by O‐related signatures captured by bulk sensitive probes, such as neutron pair distribution function (PDF), electron paramagnetic resonance (EPR), and resonant inelastic X‐ray scattering (RIXS).^[^ ^5^, ^6^, ^7^ ^]^ Of these, RIXS is regarded as the most reliable technique, with which the formation of molecular O_2_ has been observed.^[^ ^8^ ^]^ However, for the monometallic Li_2_MnO_3_, recent studies suggest that it exhibits no reversible oxygen redox,^[^ ^9^, ^10^, ^11^ ^]^ which raises issues on understanding redox mechanisms in this Ni‐ and Co‐free archetypal material.

Li_2_MnO_3_, initially prepared by Strobel et al., has an O3‐type monoclinic structure (C2/m symmetry), in which LiO_6_ and MnO_6_ octahedra stack along the *c*‐axis to form the layered structure.^[^ ^12^, ^13^ ^]^ When first being used as the cathode material for LIBs, Kalyani et al. attributed its charging capacity to the oxidation of Mn.^[^ ^14^ ^]^ However, Mn^4+^ ions in octahedral sites are unlikely to be oxidized to higher oxidation states. Later, Bruce et al. proposed the proton exchange theory, i.e., H^+^ ions intercalate into the lattice to occupy the Li^+^ vacancies and coordinate with adjacent O atoms via O─H─O bonds, resulting in a P3‐type structure (R‐3m symmetry).^[^ ^15^, ^16^, ^17^ ^]^ In contrast, Lu et al. argued that such anomalous capacity originates from the irreversible O loss.^[^ ^18^ ^]^ Indeed, both theoretical and experimental results suggested that the lattice oxygen (O^2−^) can be ultimately oxidized to molecular O_2_ and released out of the lattice.^[^ ^10^, ^19^ ^]^ For instance, by using the O K‐edge soft X‐ray absorption spectroscopy (sXAS), Oishi, and Marusczyk et al. claimed the observation of reversible oxygen redox between lattice oxygen (O^2−^) and peroxide (O_2_^2−^).^[^ ^20^, ^21^ ^]^ However, no characteristic signatures from peroxide (O_2_^2−^) were observed by the bulk‐sensitive O K‐edge RIXS yet, a quantitative study by differential electrochemical mass spectroscopy (DEMS) manifested that the amount of the released O_2_ accounted for the entire charge compensation.^[^ ^22^ ^]^ In addition, later studies by Yang et al. revealed that no spectroscopic signature of bulk oxygen activity (i.e., RIXS feature at energy loss of 7.5 eV) was detected thorough out the de‐/lithiation processes of Li_2_MnO_3_.^[^ ^11^ ^]^ Here, we adopt their definition of reversible oxygen redox, i.e., the oxidation of oxygen refers to a depopulation of oxygen electrons through the formation of electron holes or O─O dimers and the reduction of oxygen occurs for at least one cycle. In contrast, the irreversible oxygen oxidation and the subsequent release of gaseous O_2_ from the near‐surface region do not signify oxygen redox.

Notably, although it is widely believed that the pathway of forming O_2_ molecules involves the formation of electron holes on O 2p orbitals and the subsequent O─O dimerization, Van der Ven et al. proposed an alternative mechanism via theoretical calculation, i.e., the migration and oxidation of octahedral Mn^4+^ to tetrahedral Mn^7+^ followed by the spontaneous reduction of Mn and the formation of O─O dimers.^[^ ^23^, ^24^ ^]^ However, no experimental evidence supports the Mn oxidation theory at present. In summary, in the near‐surface region, the de‐lithiation of Li_2_MnO_3_ results in irreversible O_2_ release and the formation of rock‐salt or spinel‐like surfaces; in the bulk, Li_2_MnO_3_ is electrochemically inactive.

To enhance the electrochemical behaviors of Li_2_MnO_3_, introducing crystalline defects such as oxygen vacancies (V_O_) and stacking faults (SFs) has been demonstrated to be effective.^[^ ^25^ ^]^ For example, in the oxygen nonstoichiometric Li_2_MnO_3‐x_, oxygen vacancies activate the Mn sites as redox centers (Mn^3+^/Mn^4+^) for charge compensation during de‐lithiation and suppress the irreversible oxygen loss.^[^ ^26^, ^27^ ^]^ It is noteworthy that even the Mn^2+^/Mn^4+^ redox pairs can be activated via destructing the ordered structures, as demonstrated in the disordered rock‐salt (DRX) Li_2_Mn_2/3_Nb_1/3_O_2_F.^[^ ^28^ ^]^ In addition, SFs in Li_2_MnO_3_ prepared via low‐temperature syntheses are suggested to promote the formation of smooth Li percolation paths and favor the lithium extraction from the bulk, thereby resulting in higher reversible capacities.^[^ ^29^, ^30^ ^]^ However, such performance improvements mainly originate from the activation of Mn redox activities because no reversible oxygen redox activities have been observed to be involved, even in poorly crystalline samples. Recently, via in situ ^17^O nuclear magnetic resonance (NMR), Hu et al. captured reversible oxygen redox in a SF‐rich Li_2_MnO_3_ following the reversible extraction and injection of electrons in the π(Mn–O) system, in which Mn 3d and O 2p orbitals hybridize within Mn_6_ rings to collectively act as a delocalized redox center (Figure S1).^[^ ^31^, ^32^ ^]^ However, they also pointed out that once the dimerization takes place, the O─O dimers are unlikely to be reduced back to the lattice oxygen (O^2−^), consistent with previous studies.

Here, we demonstrate that, even with the formation of O─O dimers, reversible oxygen redox can be achieved in an O3‐type layered lithium manganese oxide with spinel heterostructures (LMO‐SH, Li_0.852_Mn_0.792_O_2_). The material was prepared via a unique synthetic strategy, i.e., Na^+^/Li^+^ ion exchange from a P2‐type sodium manganese oxide (P2‐NLMO) to obtain a metastable O2‐type lithium manganese oxide (O2‐LMO) and its decomposition at moderate temperatures to form an O3‐type LMO. The O2‐to‐O3 phase transformation involves the migration of Mn and the gliding of MnO_2_ slabs, resulting in spinel‐like subregions and large amounts of SFs along the *c*‐axis. Using bulk sensitive high‐resolution RIXS, we observed, for the first time, the formation and the reduction of molecular O_2_ in a Mn‐based phase over multiple cycles. The findings of this work can enrich the understanding of redox mechanisms in lithium manganese oxides and inspire the design of oxygen redox cathodes for better batteries.

## Results and Discussion

### Synthesis of LMO‐SH

**Figure 1:**
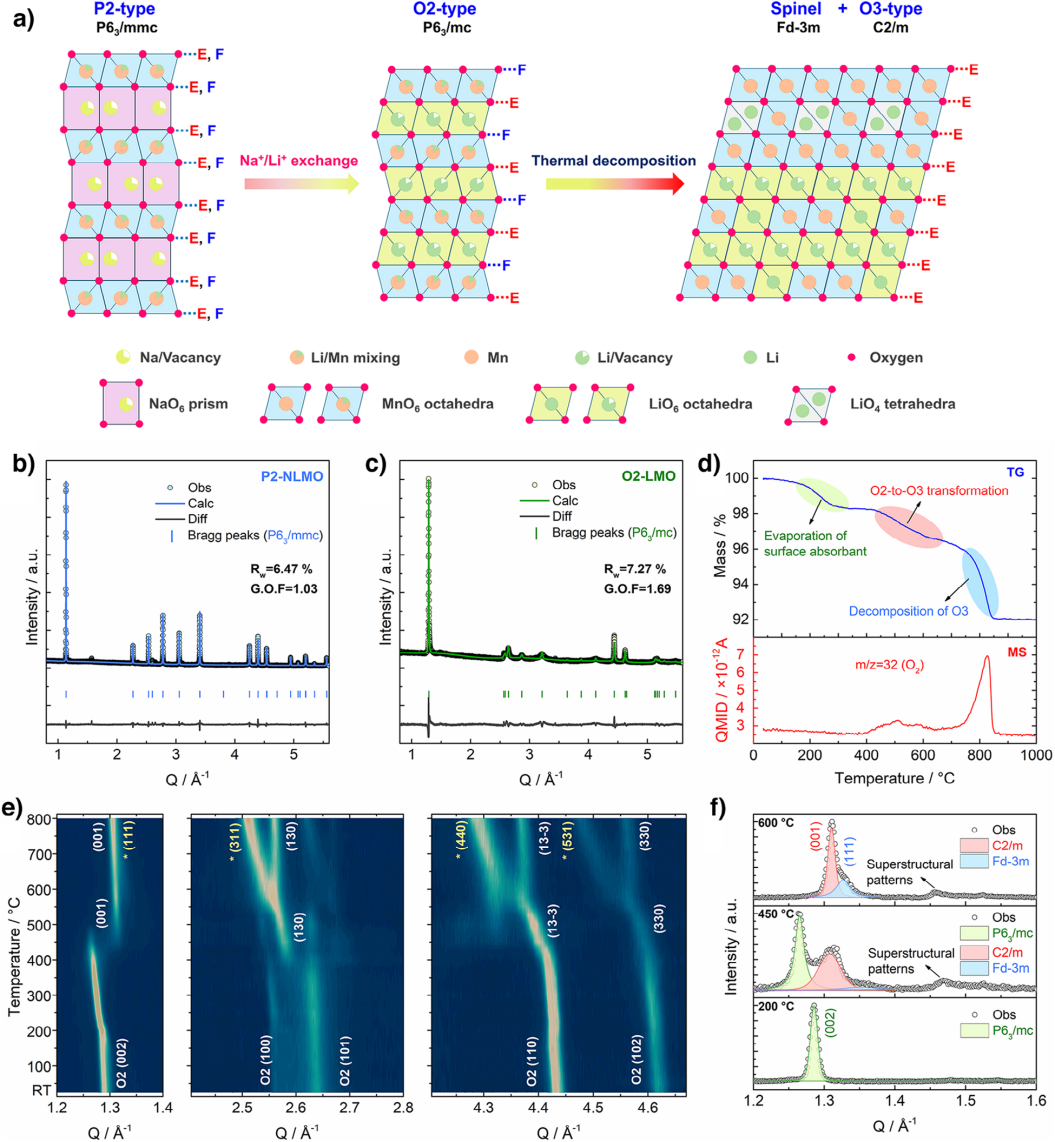
Synthesis of LMO‐SH. a) Schematic illustration of the preparation process. E and F denote edge‐sharing and face‐sharing planes, respectively. Synchrotron XRD patterns (*λ* = 0.6887 Å) of b) P2‐NLMO and c) O2‐LMO. d) TG curve (top) and quasi multiple ion detection (QMID) ion current curve collected via mass spectrometry for O_2_ (bottom). e) Contour maps of in situ VT‐XRD patterns (*λ* = 1.54 Å) for O2‐LMO. Peaks labeled by asterisks can be indexed to a spinel phase (Fd‐3m symmetry). f) Selected in situ VT‐XRD patterns at 200, 450, and 600 °C.

As illustrated in Figure 1a, the LMO‐SH was prepared via a two‐step synthetic route. First, the P2‐type Na_0.718_[Li_0.212_Mn_0.769_]O_2_ (P2‐NLMO, P6_3_/mmc symmetry) was prepared through a traditional solid reaction method,^[^ ^33^ ^]^ whose structure and composition were determined by Rietveld refinement of its synchrotron X‐ray diffraction (XRD) patterns and inductively coupled plasma‐mass spectrometry (ICP‐MS) results, respectively (details in Figure 1b and Tables S1, S2). Upon Na^+^/Li^+^ ion exchange, the P2‐type precursor transforms into an O2‐type Li_0.855_Mn_0.814_O_2_ (O2‐LMO, P6_3_/mc symmetry) via the gliding of MnO_2_ slabs (Figure 1c and Table S3).^[^ ^34^ ^]^

The as‐prepared O2‐LMO is unstable and undergoes thermal decompositions at elevated temperatures (>400 °C), as revealed by thermal gravimetric analysis‐mass spectrometry (TGA‐MS), ex situ and in situ varied temperature (VT)‐XRD results. As shown in Figure 1d, upon heating up to 400 °C, the O2‐LMO loses 1.6% weight presumably because of the evaporation of surface absorbent, while all diffraction peaks shift to lower Q due to thermal expansion of the lattice (Figures 1e, S2, and S3). In the region of 400–600 °C, along with slight O_2_ release, the O2‐LMO gradually converts to an intermediate material with predominantly an O3‐type structure but with an asymmetric first peak, which is analyzed in more detail below. This intermediate material is not stable and decomposes into a biphasic mixture along with massive O_2_ release at around 800 °C, as indicated by the emergence of new diffraction peaks.

To further understand the thermal decomposition process of the O3‐type structure, in situ VT‐XRD tests were performed during the syntheses of Li_2_MnO_3_, LiMn_2_O_4,_ and Li_0.85_Mn_0.8_O_2_ from Li_2_CO_3_ and MnCO_3_ (Figure S4). For the former two materials, only layered and spinel phases formed, respectively (Figure S4a–d, Tables S4 and S5). However, with the designed formula of Li_0.85_Mn_0.8_O_2_, at temperatures above 600 °C, its XRD patterns evolve in ways like those observed in the thermal treatment of O2‐LMO (Figure S4e), e.g., peak split in the range of Q = 2.5–2.6 Å^−1^ indicating the coexistence of layered and spinel phases. In addition, after heating the O2‐LMO at 800 °C for 2 h, its XRD patterns are consistent with those of the directly synthesized Li_0.85_Mn_0.8_O_2_ at 800 °C, which can be identified as a mixture of a layered Li_2_MnO_3_ (36.4 at%) and a spinel Li_1.29_Mn_1.71_O_4_ (63.6 at%) according to Rietveld refinement (Figure S4f and Table S6). Therefore, the thermal decomposition of O2‐LMO involves the following two steps: (1) its decomposition into an O3‐type layered phase (Li_x_Mn_y_O_2_) and a spinel phase (LiMn_2_O_4_) at moderate temperatures (400–600 °C), and (2) the subsequent decomposition of the O3‐phase to the thermally stable Li_2_MnO_3_ and spinel phases (Li_1+z_Mn_2‐z_O_4_) at higher temperatures.

$\mathrm{Step}\;1:\;{\mathrm{Li}}_{0.855}{\mathrm{Mn}}_{0.814}O_{2}\to {\mathrm{Li}}_{x}{\mathrm{Mn}}_{y}O_{2}+{\mathrm{LiMn}}_{2}O_{4}+O_{2}↑$
$\mathrm{Step}\;2:\;{\mathrm{Li}}_{x}{\mathrm{Mn}}_{y}O_{2}\to {\mathrm{Li}}_{2}\mathrm{Mn}O_{3}+{\mathrm{Li}}_{1+z}{\mathrm{Mn}}_{2-z}O_{4}+O_{2}↑$

It is noteworthy that although both P2‐NLMO and O2‐LMO have Li‐rich environments (i.e., the presence of Li within the Mn layers), as revealed by the intrinsic Raman peaks from Li–O vibrations (Figure S5),^[^ ^35^ ^]^ no superstructural peaks were observed in their XRD patterns, implying Li/Mn disordering in the Mn layers.^[^ ^36^ ^]^ However, weak superstructural patterns (i.e., peaks in the region of Q = 1.4–1.6 Å^−1^) emerge with the formation of the O3‐type layered phase (Figure 1f), manifesting the formation of ordered Li/Mn arrangements during thermal treatment.^[^ ^37^ ^]^ The LMO‐SH used in this work was prepared by heating the O2‐LMO at 500 °C for 6 h, whose chemical formula is Li_0.852_Mn_0.792_O_2_ according to ICP‐MS results.

### Structural Characterization of LMO‐SH

**Figure 2:**
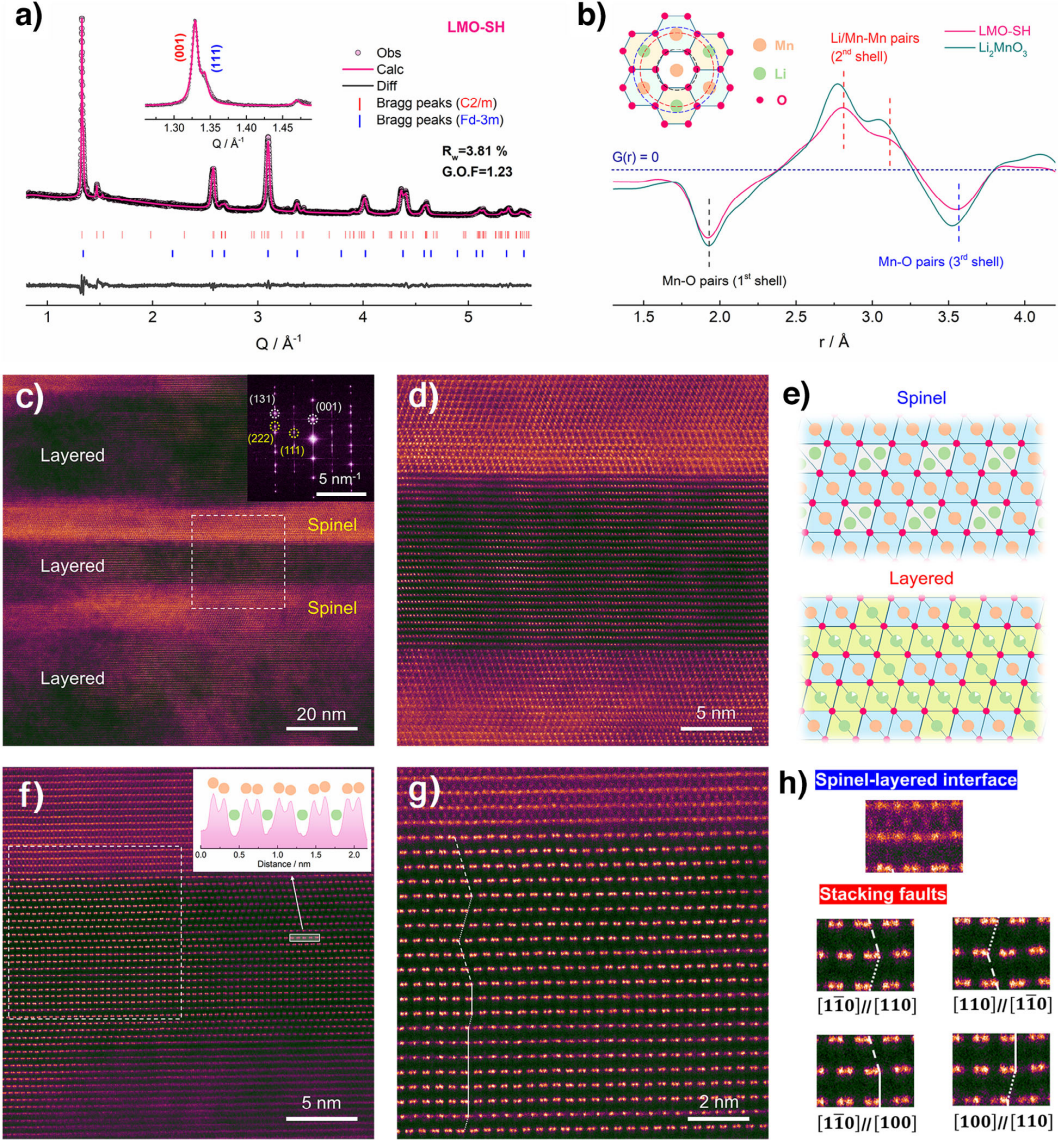
Structural characterization of LMO‐SH. a) Synchrotron XRD patterns (*λ* = 0.6887 Å) of LMO‐SH. b) Neutron PDF results of LMO‐SH and Li_2_MnO_3_. The inset displays the atom arrangement around Mn, wherein the dashed lines represent the nearest shells. c), d) HAADF‐STEM images showing spinel‐layered heterostructures along the [010] zone axis. The inset of (c) shows corresponding FFT results. (e) Illustration of spinel and layered structures. f), g) Enlarged HAADF‐STEM images along the [100] zone axis. The inset of (f) displays linear intensity profile of the marked region. h) Classification of the heterostructural interface and the SFs.

Structural analyses of the as‐prepared LMO‐SH were performed via Rietveld refinements of its synchrotron XRD patterns. First, a single O3‐type monoclinic model (C2/m, *a* = 4.9815(5), *b* = 8.5544(2), *c* = 5.0043(9) Å) was used, and the fitting is fairly good (R_w _= 5.50%, G.O.F. = 1.77, reduced χ^2 ^= 3.13, Figure S6 and Table S7). However, the shoulder over the right of the (001) peak (Q = 1.33 Å^−1^) indicates the coexistence of a second phase. Therefore, considering that the spinel phase was clearly formed at elevated temperatures, a spinel phase was added as a second phase, which improved the fitting results (R_w _= 3.81%, G.O.F. = 1.23, reduced χ^2 ^= 1.50, Figure 2a and Table S8). Therefore, the LMO‐SH (Li_0.852_Mn_0.792_O_2_) is composed of a layered phase (Li_0.887_Mn_0.771_O_2_, 95.2 at%, C2/m, *a* = 4.98103(2), *b* = 8.5511(3), *c* = 5.0099(6) Å) and a spinel phase (LiMn_2_O_4_, 4.8 at%, Fd‐3m, *a* = *b* = *c* = 8.1063(6) Å). Figure 2b shows neutron PDF results, in which some pairs have negative G(r) values because Mn has negative scattering length for neutron (−3.73 fm).^[^ ^38^ ^]^ In comparison to Li_2_MnO_3_, the layered phase in LMO‐SH has smaller lattice parameters. Therefore, although the distances between the nearest pairs (i.e., Mn‐O pairs in the 1st shell) are identical in both materials, the distances between the pairs in the 2nd and 3rd shells are smaller in LMO‐SH.

To determine whether the layered and the spinel phases are present within the same particle, spherical aberration‐corrected scanning transmission electron microscopy (AC‐STEM) was performed to investigate its structure at the atomic scale. As shown in the high angle annular dark‐field (HAADF) images, the as‐prepared material has both layered and spinel‐like regions in the bulk of a single particle (Figure 2c–e), which are also supported by the fast Fourier transform (FFT) patterns (inset of Figure 2c). After rotating the sample to the [100] zone axis (Figure 2f), typical dumbbell‐type patterns can be observed in the Mn layers (inset of Figure 2f). In addition to the spinel‐layered interfaces, SFs are identified in the enlarged STEM images of the layered phase (Figures 2g and S7), which can be classified into multiple types according to their boundaries (Figure 2h).^[^ ^39^ ^]^ As displayed in Figure S7, both the spinel‐layered heterostructures and the SFs can be observed in different regions along the [100] zone axis, while the diffuse scattering lines in the FFT image also indicate the existence of SFs (inset of Figure S7a).^[^ ^40^ ^]^ In the near‐surface regions, even the layered phase has a spinel‐type coating layer (∼ 5 nm), which can potentially mitigate side reactions with the electrolyte and suppress surface degradations (Figure S8).

### Redox Mechanisms of LMO‐SH in the 1st Cycle

**Figure 3:**
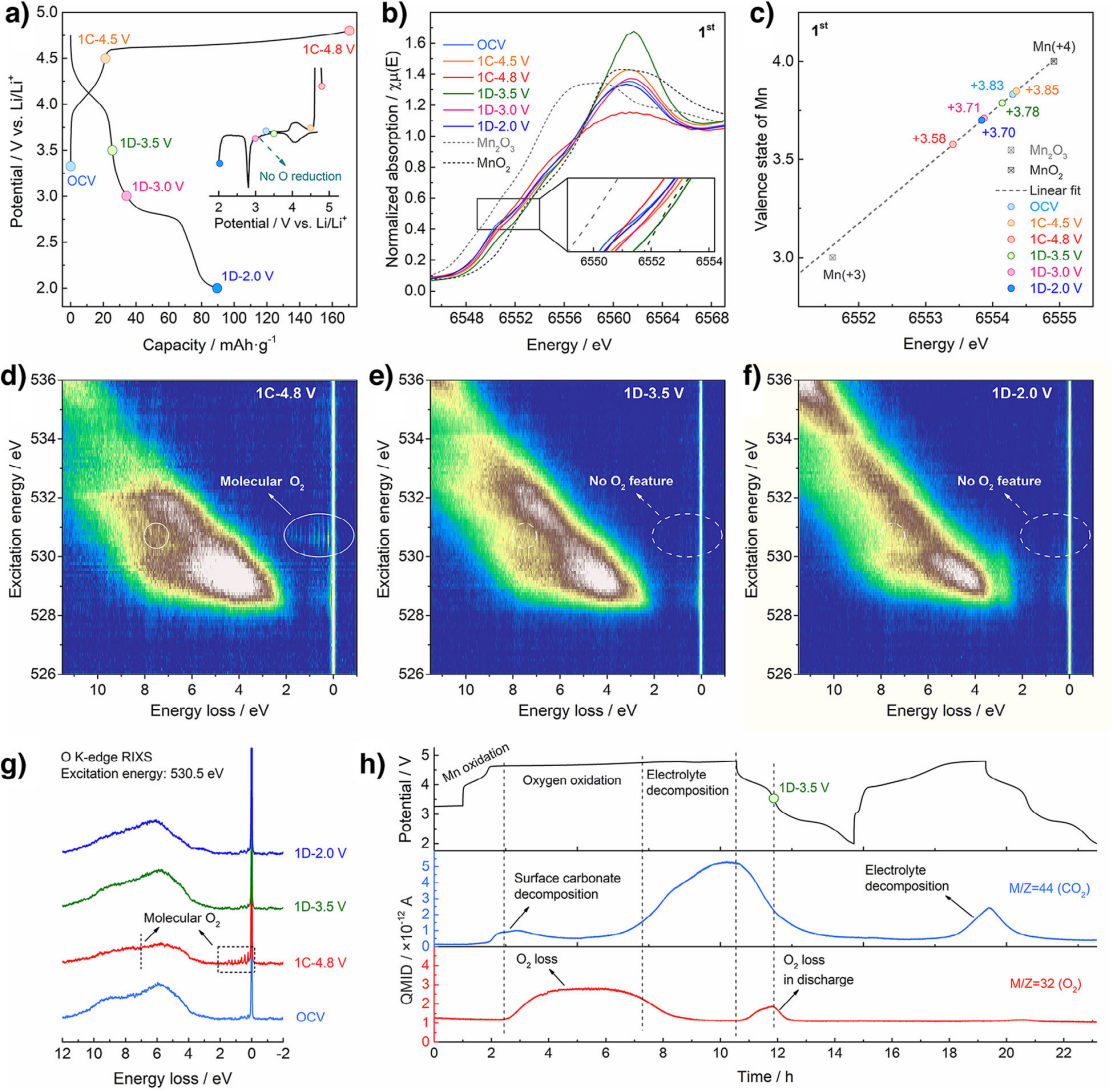
Redox mechanisms of LMO‐SH in the 1st cycle. a) Voltage profiles of the LMO‐SH electrode and corresponding *dQ/dV* curves (inset) in the 1st cycle. The colored circles mark the selected states of charge and discharge for the following characterizations. b) Mn K‐edge XANES results. c) Calculated valence states of Mn. d)–f) RIXS maps of O K‐edge at 1C‐4.8, 1D‐3.5, and 1D‐3.5 V. g) HR‐RIXS line scans at the excitation energy of 530.5 eV. h) Voltage profiles of the DEMS cell during the first two cycles (top) and QMID ion current curves collected via mass spectrometry for CO_2_ (middle) and O_2_ (bottom). In (a) and (h), the current density is 20 mA g^−1^, and the potential window is 2.0–4.8 V versus Li/ Li^+^.

To elucidate the redox mechanisms of LMO‐SH, X‐ray absorption spectroscopy (XAS) and high‐resolution resonant inelastic X‐ray scatting (HR‐RIXS) were performed at various states of charge and discharge in the 1st cycle. Figure 3a shows the voltage profiles of the LMO‐SH electrode in the 1st cycle. The initial charging profile consists of a slope and a flat plateau before and after 4.5 V, which are consistent with the removal of lithium from the spinel and the layered phases, respectively.^[^ ^41^, ^42^ ^]^ Figure 3b,c display the Mn K‐edge X‐ray adsorption near‐edge spectra (XANES) and the calculated oxidation states of Mn, respectively. The relationship between the edge position and the oxidation state was obtained via the simulation of those values for standard samples (Figure S9a,b and Table S9). It is noteworthy that the calculated oxidation state of Mn in the LMO‐SH electrode after being exposed to the electrolyte (Mn^3.83+^ at OCV; where OCV refers to open circuit voltage) is slightly lower than that of the pristine powder sample (Mn^3.99+^, Figure S9c,d). Such Mn reduction might result from the reaction between the electrode and the electrolyte to form surface carbonates,^[^ ^10^ ^]^ as evidenced by the characteristic RIXS signals from CO_3_^2−^ (Figure S10). In addition, given that LMO‐SH has Li vacancies, the hydrogenation‐driven cation reduction can take place as well, as reported for de‐lithiated cathode materials.^[^ ^43^ ^]^

Upon charging, the Mn K‐edge shifts to higher energy (OCV → 1C‐4.5 V; where C refers to charge) due to Mn oxidation and shifts to lower energy in the deeply charged state (1C‐4.5 → 1C‐4.8 V) due to an oxygen redox‐related reductive coupling mechanism (Figure S11).^[^ ^44^ ^]^ As to the O K‐edge RIXS map of the deeply charged LMO‐SH (Figure 3d), both the sharp peaks between the energy loss of 0–2 eV and the broad peak at 7.5 eV manifest the existence of trapped O_2_ molecules in the bulk.^[^ ^45^ ^]^ Recently, the characteristic RIXS signals from molecular O_2_ have also been captured in the deeply charged Li_2_MnO_3_ (4.8 V) at room temperature (293 K), yet it is not clear whether such signatures originate from electrochemical processes or beam excitation.^[^ ^46^ ^]^ In this work, all RIXS measurements were performed at 20K to minimize any possible beam damage, thereby making the spectroscopic results reliable for analyzing the intrinsic oxygen redox mechanisms.

On discharge (1C‐4.8 → 1D‐3.5 V; where D refers to discharge), the Mn K‐edge shifts to higher energy, implying that the overall oxidation state of Mn increases. During this process, the reactions in the spinel and the layered phases are suggested to be different. In the spinel phase, Mn reduction takes place. In the layered phase, the oxidized oxygen species are possibly reduced via either O_2_‐to‐O^2−^ reduction or O dominated π(Mn–O) redox, resulting in Mn oxidation. However, the O_2_‐to‐O^2−^ reduction is widely reported to be hysteretic and mainly takes place at around 3.2 V, usually identified by a broad reduction peak in *dQ/dV* curves.^[^ ^47^ ^]^ By contrast, no oxygen reduction peak is observed in the 1st cycle of the LMO‐SH electrode (inset of Figure 3a). In addition, the molecular O_2_‐related RIXS features already disappear at 1D‐3.5 V (Figure 3e). In the following 1D‐3.5 → 1D‐2.0 V process, the O K‐edge RIXS features barely change (Figure 3f,g), while the leftward shift of the Mn K‐edge manifests Mn reduction. This suggests that the trapped O_2_ molecules at the top of charge possibly have escaped from the bulk before participating in the reduction process.

To monitor the gas evolution, *operando* DEMS was employed during the initial cycles of the LMO‐SH electrode (Figure 3h). Upon the 1st charging to above 4.5 V, CO_2_ was first detected and peaked quickly due to the decomposition of surface carbonates.^[^ ^10^ ^]^ The O_2_ evolution becomes dominant over the flat plateau at around 4.6 V and decreases at the potential above 4.7 V, whereas the CO_2_ evolution dominates by the end of the charging process, presumably due to severe electrolyte decomposition. It is noteworthy that no O_2_ release was detected in the final stage. However, upon discharging to 3.5 V, O_2_ starts to release from the electrode and rises to a peak quickly, consistent with the disappearance of the spectroscopic signatures. Neither CO_2_ nor O_2_ release was detected in the following discharge to 2.0 V.

Accordingly, during the 1st charging, the lattice oxygen (O^2−^) in LMO‐SH can be oxidized to O_2_ molecules, a proportion of which are released into the electrolyte simultaneously. The rest of them are trapped in the bulk at the top of charge, which mainly escape out of the lattice at the beginning of discharge (1C‐4.8 V → 1D‐3.5 V) and cannot be reduced back to the lattice oxygen (O^2−^). As revealed by in situ XRD results (Figure S12), the initial discharge leads to fast lattice expansion, which potentially induces strain accumulation at the spinel‐layered interfaces and facilitates the release of gaseous O_2_. Therefore, in the 1st cycle, LMO‐SH exhibits partially reversible oxygen redox activities mainly via the delocalized π(Mn–O) redox mechanism, while the contribution from the reversible reaction between the lattice oxygen (O^2−^) and the molecular O_2_ is limited due to oxygen release.

### Redox Mechanisms in the 2nd Cycle and Beyond

**Figure 4:**
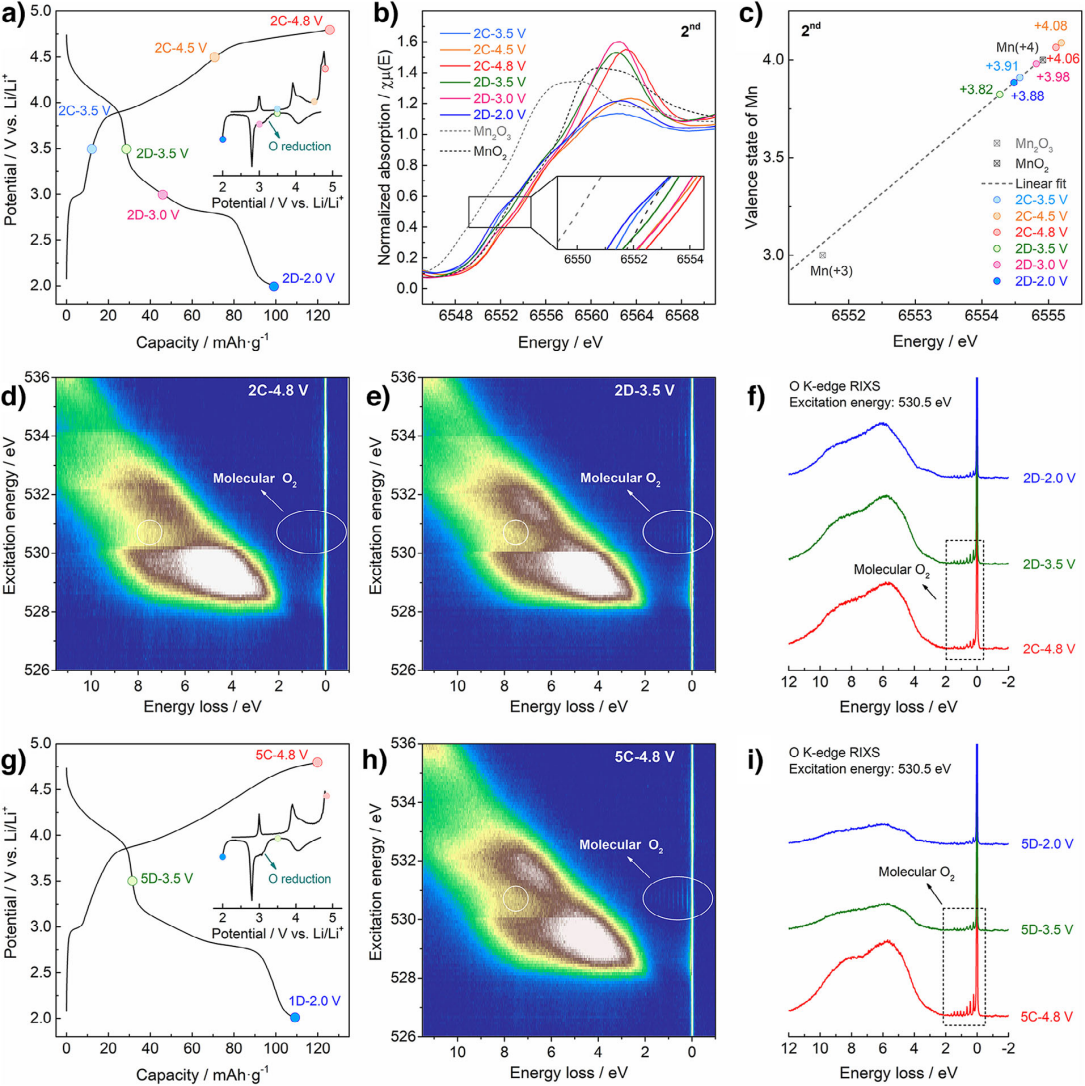
Redox mechanisms of LMO‐SH in the 2nd and 5th cycles. a) Voltage profiles of the LMO‐SH electrode and corresponding *dQ/dV* curves (inset) in the 2nd cycle. The colored circles mark the selected states of charge and discharge for the following characterizations. b) Mn K‐edge XANES results. c) Calculated valence states of Mn. d), e) RIXS maps of O K‐edge at 2C‐4.8 and 2D‐3.5 V. f) HR‐RIXS line scans in the 2nd cycle. (g) Voltage profiles of the LMO‐SH electrode and corresponding *dQ/dV* curves (inset) in the 5th cycle. h) RIXS maps of O K‐edge at 5C‐4.8 V. (i) HR‐RIXS line scans in the 5th cycle. In a) and g), the current density is 20 mA g^−1^, and the potential window is 2.0–4.8 V versus Li/ Li^+^.

To further decipher its redox mechanisms, both the Mn and O redox activities in the LMO‐SH electrode were further explored at various states of charge and discharge in the 2nd cycle, as labeled on its voltage profiles (Figure 4a). In the 2nd charge, the Mn K‐edge shifts to higher energy (2C‐3.5 → 2C‐4.5 V) and shifts slightly back to lower energy in the final stage (2C‐4.5 → 2C‐4.8 V), primarily resulting from Mn and O oxidations, respectively (Figure 4b,c). Similarly, in the deeply charged state (2C‐4.8 V), characteristic RIXS signals from O_2_ can be observed, manifesting the existence of trapped O_2_ (Figure 4d).

In the 2nd discharge, the Mn K‐edge shifts to lower energy in the 2C‐4.8 → 2D‐3.5 V process, meaning that the overall oxidation state of Mn decreases. Therefore, during this process, while π(Mn‐O) redox might occur in the layered phase, Mn reduction in the spinel phase is suggested to be dominant. Specifically, unlike the disappearance of O_2_ caused by massive oxygen release, the molecular O_2_‐related RIXS features remain at 2D‐3.5 V (Figure 4e), meaning that the O_2_ molecules are still trapped in the bulk and making their reduction back to the lattice oxygen possible.

In the following discharge, the Mn K‐edge shifts to higher energy (2D‐3.5 → 2D‐3.0 V) and lower energy (2D‐3.0 → 2D‐2.0 V), indicating the oxidation and the reduction of Mn, respectively. Meanwhile, the characteristic RIXS signals from O_2_ become insignificant in the deeply discharged state (2D‐2.0 V, Figure 4f). Given that no significant O_2_ release was detected in the 2D‐3.5 V → 2D‐2.0 V process, both the Mn oxidation and the disappearance of O_2_ molecules reconcile with the O_2_‐to‐O^2−^ reduction. In addition, a broad oxygen reduction peak can be spotted in the 2nd *dQ/dV* curves (inset of Figure 4a).

Combined, this suggests that, in the 2nd cycle of LMO‐SH, the lattice oxygen (O^2−^) can be oxidized to O_2_ molecules upon charging, which are mainly trapped in the bulk at the top of charge and reduced back to lattice oxygen (O^2−^) in discharge. Moreover, both the electrochemical and spectroscopic features are observed in the 5th cycle (Figure 4g–i), meaning that such reversible oxygen redox is stable in LMO‐SH. Given that the layered Li_2_MnO_3_ has been widely reported to be electrochemically inactive and exhibits no reversible oxygen redox, this work marks the first experimental observation of reversible oxygen redox between lattice oxygen (O^2−^) and molecular O_2_ in a Mn‐based layered phase without other transition metals (e.g., Ni and Co).

### Role of the Spinel Heterostructure in LMO‐SH

To understand the unique properties of LMO‐SH, two structural features should be considered, i.e., the stacking fault and the spinel‐layered heterostructure. Via low temperature syntheses, plenty of SFs‐rich Li_2_MnO_3_ samples have been reported,^[^ ^29^, ^48^ ^]^ which usually deliver higher capacities. However, such performance improvements mainly originate from higher degrees of layered‐to‐spinel/ rock‐salt transformations, while the converted phases undergo reversible de‐/lithiation via cation redox mechanisms.^[^ ^49^ ^]^ Although some claimed that SFs can enable reversible oxygen redox via a π‐hybridization mechanism,^[^ ^31^ ^]^ no reversible oxygen redox between the lattice oxygen (O^2−^) and molecular O_2_ has been observed even in SF‐rich Li_2_MnO_3_.^[^ ^11^ ^]^ Therefore, we believe that the spinel heterostructure plays a critical role in determining the properties of LMO‐SH.

**Figure 5:**
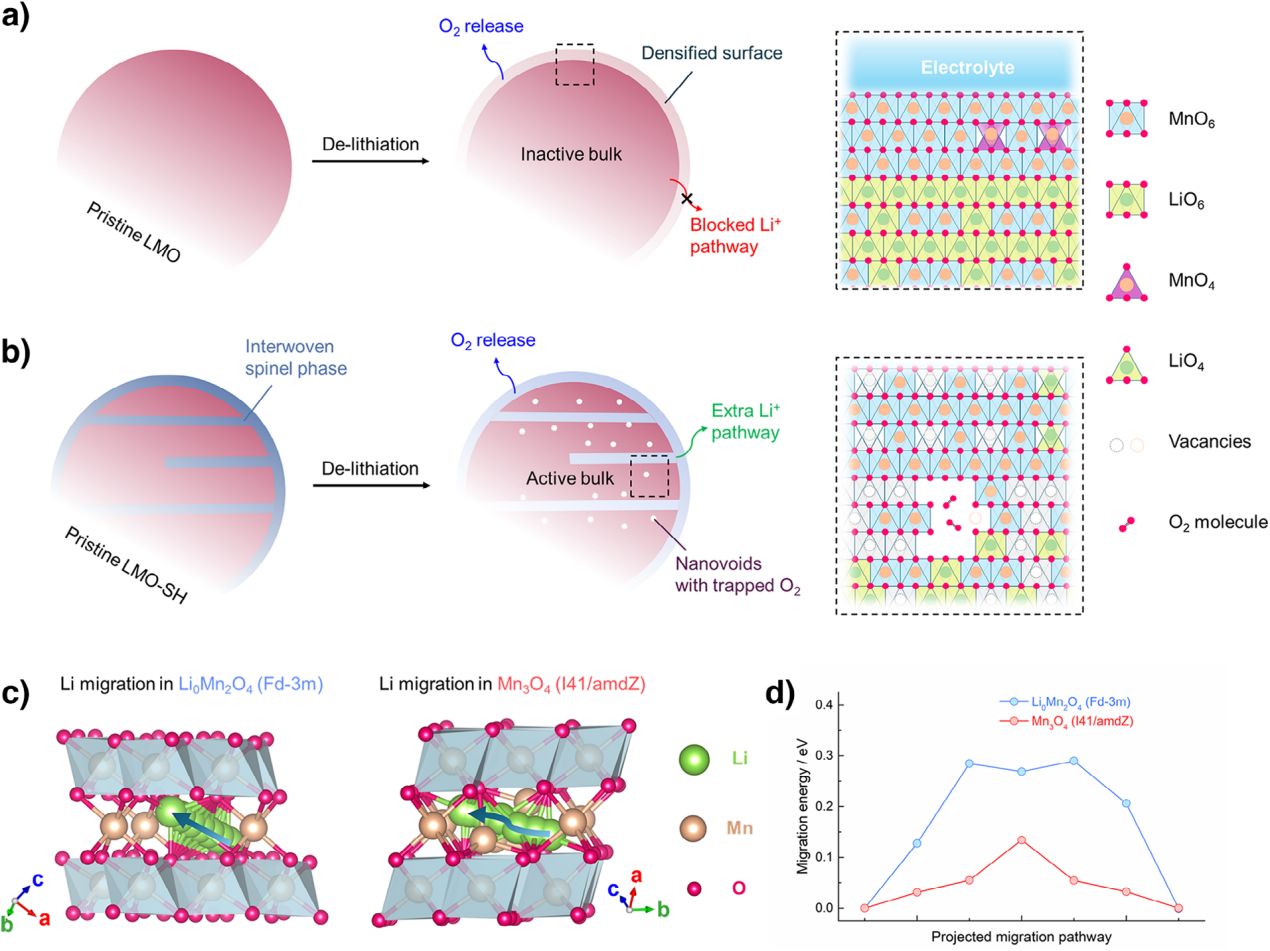
Schematic illustration of reversible oxygen redox in LMO‐SH. a) Structural evolution in typical Li_2_MnO_3_. The initial de‐lithiation leads to O_2_ release and surface densification. The densified surface blocks the Li^+^ percolation and results in an electrochemically inactive bulk. b) Structural evolution in LMO‐SH. The interwoven spinel structure provides an extra Li^+^ pathway to activate the bulk. The formed O_2_ molecules can be trapped in nanovoids and be reduced back to lattice oxygen. The insets on the right illustrate the structures of the regions marked by the dashed rectangles. c) Li migration pathways in two kinds of spinel structures, i.e., Li_0_Mn_2_O_4_ (Fd‐3m, left) and Mn_3_O_4_ (I41/amdZ, right). The green, brown, and pink balls represent Li, Mn and O atoms, respectively. The blue arrows indicate trajectories of the Li jump between two adjacent tetrahedral sites with an intermediate octahedral site. d) Relative migration energies along the projected migration pathways of Li ions.

As illustrated in Figure 5a, removing Li^+^ ions out of the layered Li_2_MnO_3_ (LMO) usually leads to irreversible O_2_ release and the formation of densified rock‐salt/ spinel surfaces.^[^ ^50^, ^51^ ^]^ The accumulation of those Li‐free phases can block the Li^+^ pathway and make the bulk electrochemically inactive.^[^ ^52^ ^]^ Therefore, the LMO electrode delivers very low capacities (Figure S13a, i.e., <15 mAh g^−1^). In the Nyquist plots of the LMO electrode (Figure S13b), after 5 cycles, the new semicircle in the middle frequency region represents the charge transfer resistance (R_ct_) in newly formed phases caused by surface degradations, leading to higher resistances (R_sf _+ R_ct_). By contrast, for the LMO‐SH electrode, the surface resistance (R_sf_) decreases after 5 cycles, suggesting that the spinel coating layer can protect the surface from degradation (Figure S13c). In addition, the interwoven spinel phase provides an extra pathway to further extract Li^+^ ions from the bulk of the layered phase (Figure 5b).

First‐principles calculations were performed to elucidate the energetics of the Li migrations in two kinds of spinel structures. As schematically illustrated in Figure 5c, Li ions jump along a linear trajectory in the de‐lithiated Li_0_Mn_2_O_4_ (Fd‐3m symmetry) and a zigzag trajectory in Mn_3_O_4_ (I41/amdZ symmetry), which have energy barriers of 0.29 and 0.13 eV, respectively (Figure 5d). Considering that Li migration is more difficult in densified rock‐salt structures (i.e., > 0.5 eV as previously reported),^[^ ^53^ ^]^ the spinel heterostructure can boost the Li migration and electrochemically activate the bulk of LMO‐SH. In addition, thanks to the rigid framework, O_2_ molecules generated via oxygen oxidation can be trapped in the bulk and reduced back to lattice oxygen in discharge, thereby achieving reversible oxygen redox.

## Conclusion

In summary, we report a Mn‐based layered oxide with spinel heterostructures (LMO‐SH), which demonstrates reversible oxygen redox reactions between the lattice oxygen (O^2−^) and molecular O_2_. Although Li_2_MnO_3_ (LMO) is believed to be the anionic redox active component in LRMOs, the prevailing understanding is that it exhibits no reversible oxygen redox in the absence of other transition metals (e.g., Ni and Co). Recent studies indicate that introducing SFs into LMO can partially activate its reversible oxygen redox activity via the delocalized π(Mn–O) system. However, even in SF‐rich LMO, once the O─O dimers form, they are more likely to be released as gaseous O_2_ rather than be reduced back to the lattice. By contrast, in LMO‐SH, the layered‐spinel heterostructure not only provides a fast Li^+^ pathway to active the bulk but also acts as a rigid framework to confine the formed O_2_ molecules, thereby making their reduction back to the lattice oxygen (O^2−^) possible. Therefore, considering that only Mn exists in LMO‐SH, the findings of this study can deepen the mechanistic understanding of lithium manganese oxides while offering new design principles for developing lithium‐rich cathode materials with stable and reversible oxygen redox activities.

Furthermore, this work establishes an innovative defect engineering strategy to integrate spinel heterostructures in layered oxide architectures. Introducing defects in the bulk or in the near‐surface regions has been demonstrated to be effective in improving the properties of LMO. For instance, SFs and oxygen vacancies can activate the redox activities in the bulk. Nevertheless, SFs may slow down ionic transport kinetics,^[^ ^54^ ^]^ while oxygen vacancies induce lattice distortion and structural failures,^[^ ^55^ ^]^ thereby resulting in fast performance degradations. From the perspective of surface modification, constructing spinel coating layers can mitigate undesirable interface reaction, provide fast Li^+^ pathways, and prevent oxygen release.^[^ ^56^, ^57^ ^]^ However, the intrinsic properties of the bulk are unlikely to be changed via surface modification.

Herein, in LMO‐SH, the spinel structure is successfully embedded into the bulk of the layered LMO, which acts as a rigid framework to stabilize the layered structure and provides fast Li^+^ pathways to activate electrochemical processes in the bulk. Meanwhile, a spinel coating layer forms simultaneously, which can stabilize the surface crystal structure. In addition, considering that the layered‐spinel heterostructure is formed via the thermal decomposition of a metastable O2‐type LMO, both the composition and the ratio of the spinel phase can be tuned via changing the treating temperature or the amount of Li in the O2‐type precursors. Given the enhanced structural resilience imparted by these heterostructures–particularly against lattice oxygen loss and phase degradation–this defect engineering approach establishes a generalizable paradigm for developing robust, high‐capacity electrode materials.

## Supporting Information

Experimental section, Figures S1–S13, Tables S1–S9, and supplementary references.

## Conflict of Interests

The authors declare no conflict of interest.

## Supporting information



Supporting Information

## Data Availability

The data that support the findings of this study are available from the corresponding authors upon reasonable request.
