# Supplementary material for: Spinel‐Layered Heterostructure Enables Reversible Oxygen Redox in Lithium Manganese Oxide
Source: Angew Chem Int Ed Engl. 2025 Jul 11;64(36):e202511054. doi: 10.1002/anie.202511054 (PMC12402837; doi:10.1002/anie.202511054)
Supplement: Supplementary file 1 — Supporting Information [file ANIE-64-e202511054-s001.docx]

Spinel-Layered Heterostructure Enables Reversible Oxygen Redox in Lithium Manganese Oxide

Yanfang Wang,^[a,b,c]^ Cheng Li,^[d]^ Yulin Cao,^[a]^ Juping Xu,^[e,f]^ Dominic Gardner,^[b,g]^ Wilgner Lima da Silva,^[b,g]^ Yongcong Huang,^[a]^ Fangchang Zhang,^[a]^ Mingzhou Li,^[a]^ Yingzhi Li,^[a]^ Wen Yin,^[e,f]^ Kaili Zhang,*^[c]^ Phoebe K. Allan,*^[b,d]^ and Zhouguang Lu*^[a]^

[a] Dr. Y. F. Wang, Y. L. Cao, Y. C. Huang, F. C. Zhang, M. Z. Li, Dr. Y. Z. Li, Prof. Z. G. Lu
Department of Materials Science and Engineering
Southern University of Science and Technology
Shenzhen, 518055, China
E-mail: zglu@sustech.edu.cn

[b] Dr. Y. F. Wang, D. Gardner, Dr. W. L. Silva, Prof. P. K. Allan
School of Chemistry
University of Birmingham
Edgbaston, Birmingham, B15 2TT, UK
E-mail: p.allan@bham.ac.uk

[c] Dr. Y. F. Wang, Prof. K. L. Zhang
Department of Mechanical Engineering
City University of Hong Kong
Tat Chee Avenue, Kowloon Tong, Hong Kong SAR, China
E-mail: kaizhang@cityu.edu.hk

[d] Dr. C. Li
Eastern Institute for Advanced Study
Eastern Institute of Technology
Ningbo, Zhejiang, China

[e] Dr. J. P. Xu, Prof. W. Yin
Chinese Academy of Sciences
Institute for High Energy Physics
Beijing, 100049, China

[f] Dr. J. P. Xu, Prof. W. Yin
Spallation Neutron Source Science Centre
Dongguan, 523803, China

[g] D. Gardner, Dr. W. L. Silva, Prof. P. K. Allan
The Faraday Institution
Harwell Campus, Didcot, UK

This file includes Experimental Section, Figure S1-S13, Table S1-S9, and Supplementary References.

**Experimental Section**

**Materials syntheses.** To prepare the P2-type precursor (NLMO), manganese carbonate (MnCO_3_, Aladdin, ≥ 99.95 %) was mixed with lithium carbonate (Li_2_CO_3_, Aladdin, ≥ 99.99 %) and sodium carbonate (Na_2_CO_3_, Aladdin, ≥ 99.5 %) in a molar ratio of 8: 1.05: 4.2. Then, the mixture was ground by hand and annealed in a muffle furnace. The programmed heating process includes a water removal step at 450 °C for 5 h (5 °C/ min), an annealing period at 800 °C for 12 h (2°C/ min) and a cooling period to 100 °C (2 °C/ min) in air. To obtain the O2-LMO, the P2-type precursor was mixed with LiNO_3_ (Aladdin, ≥ 99.9 %) and LiCl (Aladdin, ≥ 99.99 %) in a weight ratio of 1: 8.2 :1.8 and annealed in a muffle furnace at 280 °C for 4 h in air. The as-obtained product was washed thoroughly with distilled water and dried at 80 °C overnight. Finally, to get the LMO-SH, the dried O2-LMO was annealed at 500 °C for 6 h in air. The O3-type Li_2_MnO_3_, spinel LiMn_2_O_4_ and Li_0.85_Mn_0.8_O_2_ were synthesized via traditional solid-state reactions. The programmed heating process includes a water removal step at 450 °C for 5 h (5 °C/ min), an annealing period at 800 °C for 12 h (2°C/ min) and a cooling period to 100 °C (2 °C/ min) in air.

**General characterizations.** Powder X-ray diffraction (XRD) and *in situ* XRD tests were performed on a Rigaku X-ray diffractometer (Rigaku, Japan) with a Cu Kα radiation (λ=1.54 Å, 9 kW). Synchrotron XRD patterns were collected on BL14B (E=18 KeV, λ=0.6887 Å) at Shanghai Synchrotron Radiation Facility (SSRF). Rietveld refinement was performed by using GSAS-II.^1^ Neutron pair distribution function (PDF) data were collected on Multi-Physics Instrument (MPI) at the China Spallation Neutron Source (CSNS).^2^ Approximately 1 g of powder was placed into a vanadium-nickel alloy sample cell (Φ=9mm) to collect total neutron scattering data. The data were processed with the Mantid software, to correct for absorption, inelasticity effects, multiple scattering, and background. The pair distribution function G(r) was then obtained by Fourier transforming the corrected total scattering structure function S(Q) with a maximum momentum Q_max_ of 31.5 Å^-1^. Raman spectra were collected on a Renishaw inVia Raman spectrometer. Scanning transmission electrode microscopy (STEM) images were collected by using spherical aberration corrected transmission electron microscope (ACTEM) (Titan Themis G2). Thermal gravimetric analysis-mass spectrometry (TGA-MS) test was performed on STA449F3-QMS403D (Netzsch, Germany).

***In situ* VT-XRD.** *In situ* varied temperature (VT)-XRD tests were performed on a Rigaku X-ray diffractometer (Rigaku, Japan) with a Cu Kα radiation (λ=1.54 Å, 9 kW) and a one-dimensional detector was used to increase the intensity of diffractive signals. Specifically, for the O2-LMO powder sample, it was placed on a platinum holder and transferred into the high-temperature chamber. To monitor the structural evolutions during the syntheses of Li_2_MnO_3_, LiMn_2_O_4_ and Li_0.85_Mn_0.8_O_2_, the designed ratios of MnCO_3_ and Li_2_CO_3_ were ground by hand and pressed into thin tablets (~100 mg), which were placed on a Al_2_O_3_ holder and transferred into the high-temperature chamber. All samples were heated from room temperature (R.T., 25 °C) to 800 °C (5 °C/ min) with a holdup time of 30 minutes for every 25 °C to collect XRD patterns.

**Cell assembly and electrochemistry.** The as-prepared LMO-SH, Super P carbon and polyvinylidene fluoride (PVDF) in the weight ratio of 8: 1: 1 were mixed homogeneously in N-methyl pyrrolidone (NMP) and coated on an aluminum foil (12 μm in thickness) before cutting it into pieces (1.2 cm in diameter). CR2016-type coin cells were assembled in an argon-filled glovebox by using the as-prepared electrodes (cathode, ~50 μm in thickness), lithium metal plates (anode, 200 μm in thickness) and Celgard membranes (separator, 25 μm in thickness). The areal mass loading of active material was around 2.0 mg/ cm^2^. The electrolyte was a solution of 1 M LiPF_6_ in ethylene carbonate (EC)/ dimethyl carbonate (DMC) binary solvent (1:1 in volume). The amount of electrolyte injected for each coin-cell was around 40 μL in volume. Electrochemical tests were performed on the Neware battery cycler (CT-4008-5V-10mA-164, Shenzhen, China). All tests were performed at 25 °C.

***Operando* DEMS.** The differential electrochemical mass spectroscopy (DEMS) test was performed on a mass spectrometry system (QAS100-Li, Linglu, China) with a commercial quadrupole mass spectrometer (Thermo Fischer) and a turbomolecular pump (Pfeiffer Vacuum). The operando mass spectrometry cell consisted of a lithium metal anode disk, a separator (Whatman GF/A), and a cathode disk composed of 80 wt % active material, 10 wt% Super P carbon, and 10 wt % polyvinylidene fluoride (PVDF) binder. The electrolyte was a solution of 1 M LiPF_6_ in ethylene carbonate (EC)/ dimethyl carbonate (DMC) binary solvent (1:1 in volume). The amount of electrolyte injected for each coin-cell was around 200 μL in volume. The areal mass loading of active material was around 10.0 mg/ cm^2^.

**XAS and HR-RIXS.** The Mn K-edge XANES spectra were collected at beamline 11B of Shanghai Synchrotron Radiation Facility (SSRF). The XAS energy was calibrated by the first inflection point of the Mn K-edge of a metallic Mn foil (6539 eV), which was measured simultaneously in each scan. To prepare LMO-SH electrodes at different electrochemical states, the above-mentioned coin cells were dis-/charged to different voltages and maintained at corresponding voltages for 30 min, before being disassembled in the argon protected glove box, washed with dimethyl carbonate (DMC) and naturally dried in argon flow. High resolution resonant inelastic X-ray scattering (HR-RIXS) for the O K-edge were collected at the I21 beamline at Diamond Light Source.^3^ Samples were transferred to the spectrometer using a vacuum-transfer suitcase to avoid air exposure and were pumped down to ultra-high vacuum (UHV) and left to fully degas overnight. RIXS maps were measured in 0.2 eV energy increments from 526 eV to 536 eV at five different sample locations summed together. RIXS line scans were recorded at the resonance energy for molecular O_2_ (530.5 eV) at 15 different sample locations and averaged together. All data were obtained in partial fluorescence mode for bulk sensitivity. All measurements were performed at 20 K to minimize any possible beam damage. The energy loss was calibrated by the elastic scattering peak (∆E = 0 eV).

**First-principles calculations.** The spin-polarized density functional theory (DFT) calculations were carried out in the Vienna ab initio simulation package (VASP).^4^ All the DFT energies were estimated within the generalized gradient approximation (GGA) parameterization using the Perdew-Burke-Ernzerhof (PBE) functional.^5^ Effective Hubbard-*U* parameter of 3.9 was applied to the 3*d* electrons of Mn.^6^ The van der Waals correction of Grimme’s DFT-D3 model was adopted, and the climbing image nudged elastic band method was used to compute the transition states.^7,8^ For the de-lithiated Li_0_Mn_2_O_4_ model, a unit cell with parameters of a = 8.1 Å, b = 8.1 Å, and c = 8.1 Å was used with a 3×3×3 gamma-point-centered *k*-point mesh. For the Mn_3_O_4_ model, a unit cell with parameters of a = 11.5 Å, b = 11.5 Å, and c = 9.4 Å was used with a 2×2×3 gamma-point-centered *k*-point mesh. The energy cutoff was set to be 450 eV. The structures were fully relaxed until the interatomic forces were smaller than 0.02 eV/Å, and the energy convergent standard was 10^-5^ eV.

Table S1. **ICP-MS results**

|  | **7 Li [He]** | | **23 Na [He]** | | **55 Mn [He]** | |
| --- | --- | --- | --- | --- | --- | --- |
|  | Conc. [ppb] | Conc. RSD | Conc. [ppb] | Conc. RSD | Conc. [ppb] | Conc. RSD |
| **P2-NLMO** | 162.65(3) | 9.29(1) | 1808.98(3) | 0.76(3) | 4633.35(4) | 1.94(3) |
| **O2-LMO** | 775.29(2) | 1.13(1) | N/A | N/A | 5808.90(5) | 1.31(8) |
| **LMO-SH** | 736.71(5) | 2.13(5) | N/A | N/A | 5401.57(3) | 1.21(4) |

Table S2. Rietveld refinement parameters for P2-NLMO

| **P2-Na_0.718_[Li_0.212_Mn_0.769_]O_2_** Space group: P 6_3_/mmc | | | | | | |
| --- | --- | --- | --- | --- | --- | --- |
| Lattice parameters:  a = 2.8600(1) Å, b = 2.8600(1) Å, c = 11.0633(2) Å; α = 90°, β = 90°, γ = 120°; V_unit cell_ = 78.37(1) Å^3^; | | | | | | |
| Atom | Fractional coordinates | | | Multiplicity | Occupancy | U_iso_ |
|  | x | y | z |  |  |  |
| Na1 | 0 | 0 | 0.25 | 2 | 0.359 | 0.028(1) |
| Na2 | 1/3 | 2/3 | 0.75 | 2 | 0.359 | 0.028(1) |
| Li | 0 | 0 | 0 | 2 | 0.212 | 0.028(2) |
| Mn | 0 | 0 | 0 | 2 | 0.769 | 0.028(2) |
| O | 1/3 | 2/3 | 0.0821(3) | 4 | 1 | 0.032(4) |
| Refinement results: R_w_ = 6.47 %, G.O.F. = 1.03, reduced χ^2^ = 1.07 | | | | | |  |

Table S3. Rietveld refinement parameters for O2-LMO

| **O2-Li_0.669_[Li_0.186_Mn_0.814_]O_2_** Space group: P 6_3_/mc | | | | | | |
| --- | --- | --- | --- | --- | --- | --- |
| Lattice parameters:  a = 2.8311(2) Å, b = 2.8311(2) Å, c = 9.7217(3) Å; α = 90°, β = 90°, γ = 120°; V_unit cell_ = 67.48(2) Å^3^; | | | | | | |
| Atom | Fractional coordinates | | | Multiplicity | Occupancy | U_iso_ |
|  | x | y | z |  |  |  |
| Li1 | 1/3 | 2/3 | 0.2410 | 2 | 0.335 | 0.025(1) |
| Li2 | 0 | 0 | 0.2600 | 2 | 0.334 | 0.025(1) |
| Li3 | 2/3 | 1/3 | 0 | 2 | 0.186 | 0.025(1) |
| Mn | 2/3 | 1/3 | 0 | 2 | 0.814 | 0.025(1) |
| O1 | 2/3 | 1/3 | 0.4091(1) | 2 | 1 | 0.026(7) |
| O2 | 0 | 0 | 0.1387(6) | 2 | 1 | 0.026(7) |
| Refinement results: R_w_ = 7.27 %, G.O.F. = 1.69, reduced χ^2^ = 2.87 | | | | | |  |


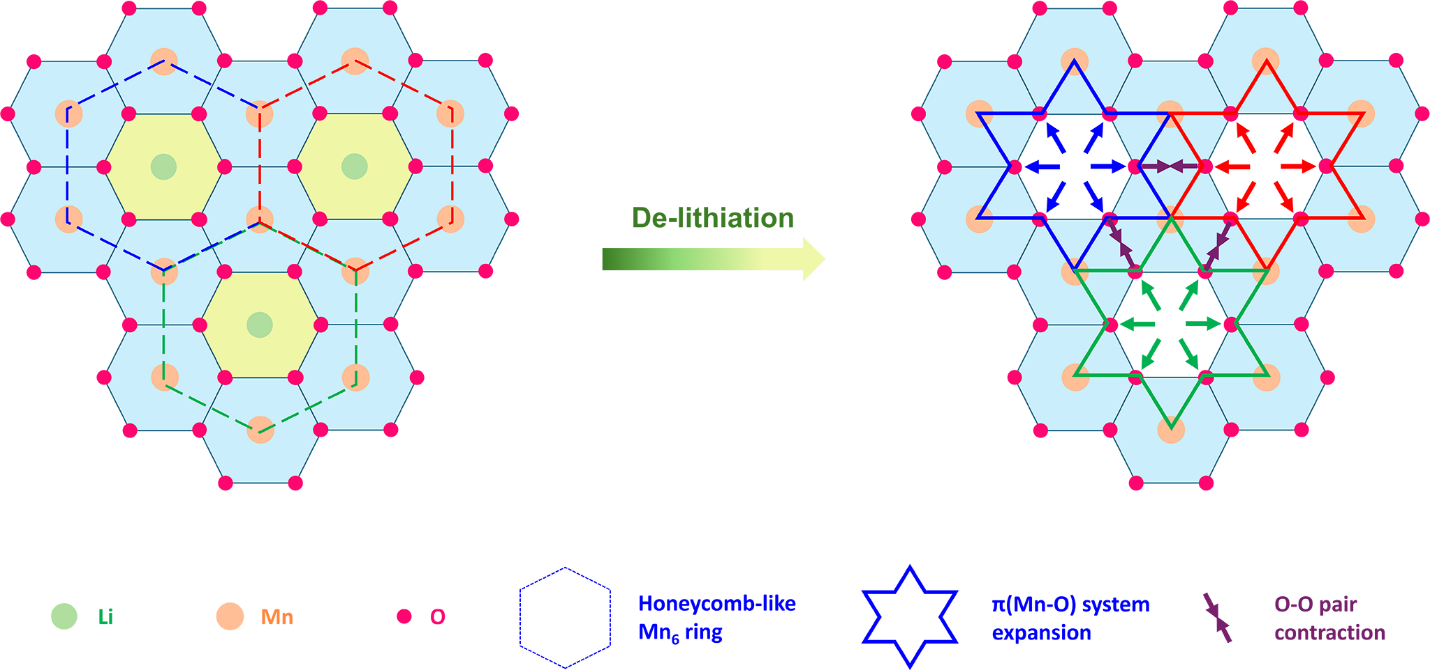


**Figure S1.** Schematic illustration of the delocalized π(Mn-O) system in Li_2_MnO_3_.

Delocalized π-redox is a recently proposed theory to explain the charge compensation mechanisms in cathode materials, which connects traditional transition metal redox with the lattice oxygen redox behaviors. Traditionally, upon charging and discharging, transition metal (TM) ions can be oxidized and reduced for charge compensation, respectively. On the other hand, many studies have revealed that the lattice oxygen (O^2-^) can participate in redox reactions, resulting in the formation of localized O-holes, O-O dimers and even molecular O_2_. In both scenarios, the redox active centers are localized on specific orbitals, i.e., TM d orbital (TM-centric) or O p orbital (O-centric), resulting in changes of atomic oxidation states.

In π-redox systems, the redox-active orbital is delocalized between multiple transition metal and oxygen atoms, meaning that the process can no longer be described in terms of atomic oxidation states, but rather as a hybridized redox center over an extended molecular unit. Specifically, in the case of Li_2_MnO_3_, each Li is surrounded by six Mn in the TM layers, i.e., honeycomb superstructure. Upon the removal of Li, both Mn 3d and O 2p orbitals contribute partially to charge compensation, resulting in an electrochemically active π-state delocalized within the Mn_6_ rings. During this process, the Mn_6_ rings expand and the O-O pairs contract (**Figure S1**). Therefore, the π(Mn-O) system is a hybridized network of Mn 3d and O 2p orbitals, which collectively acts as a delocalized redox center.


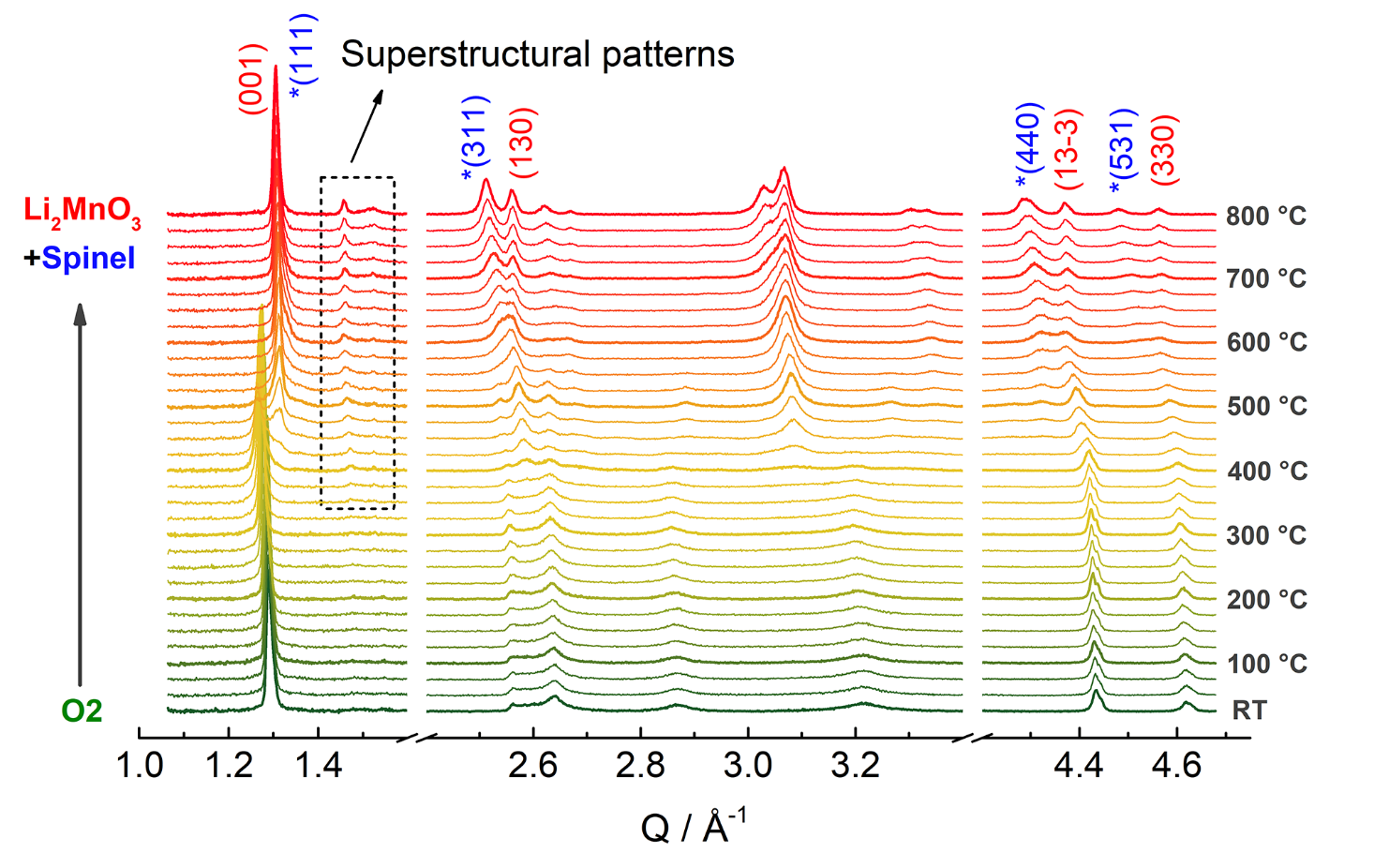


**Figure S2.** *In situ* VT-XRD patterns (λ=1.54 Å) for O2-LMO. Peaks labeled by asterisks can be indexed to a spinel phase (Fd-3m symmetry).

At room temperature (R.T.), the XRD peak at around Q= 1.30 Å^-1^ corresponds to the (002) peak of the O2-phase. In the region of R.T. to 300 °C, the O2-(002) peak shifts to lower Q due to thermal expansion. Further heating up to 400 °C induces phase transformation and produces a material with a different symmetry. Such an intermediate phase decomposes into two phases at higher temperatures (> 600 °C), as indicated by the emergence of new diffraction peaks.


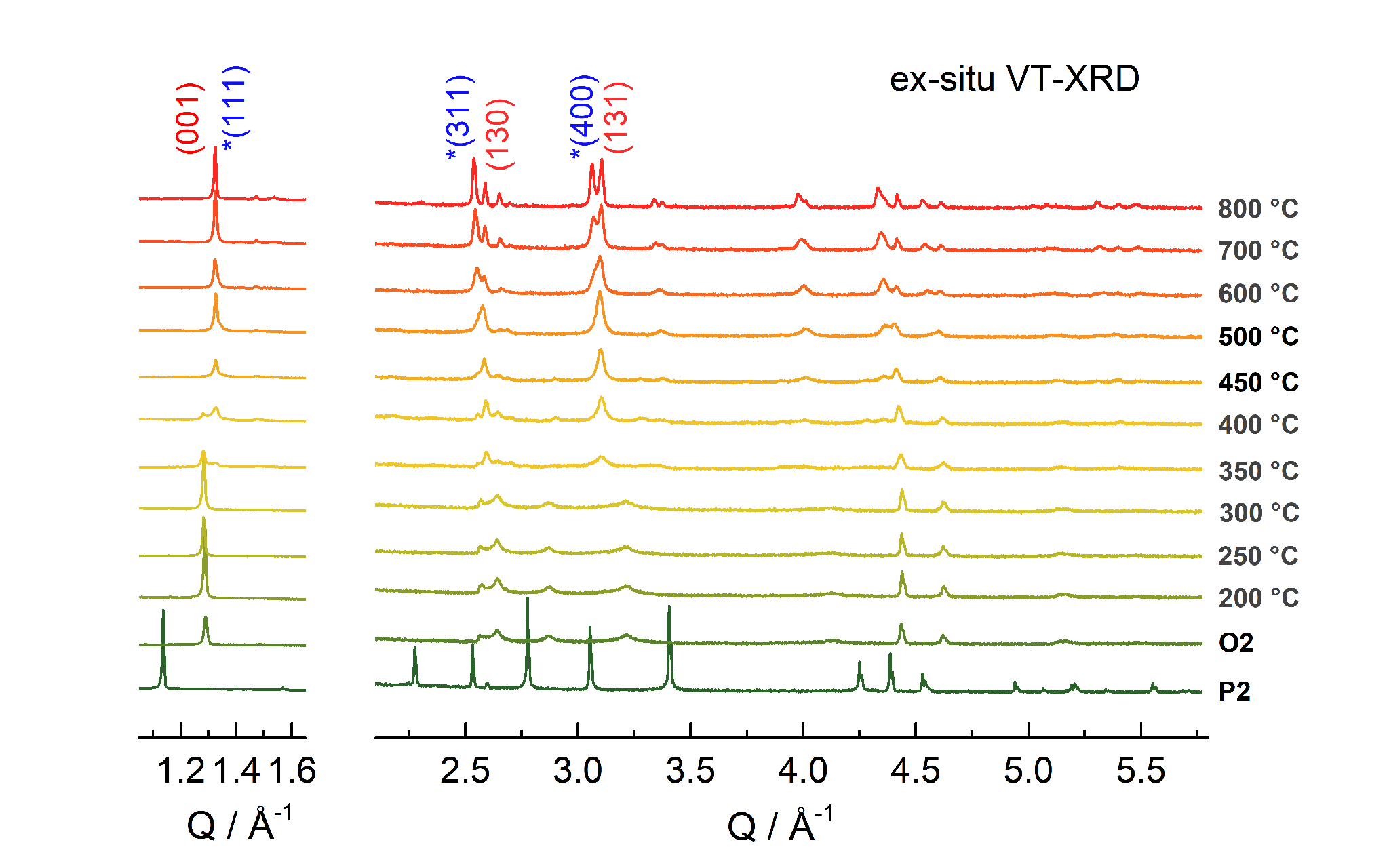


**Figure S3.** *Ex situ* VT-XRD patterns (λ=1.54 Å) for O2-LMO. Peaks labeled by asterisks can be indexed to a spinel phase (Fd-3m symmetry). For each sample, the as-prepared O2-LMO was heated at a specific temperature in air for 2 h. The bottom line represents XRD patterns of the P2-NLMO precursor.


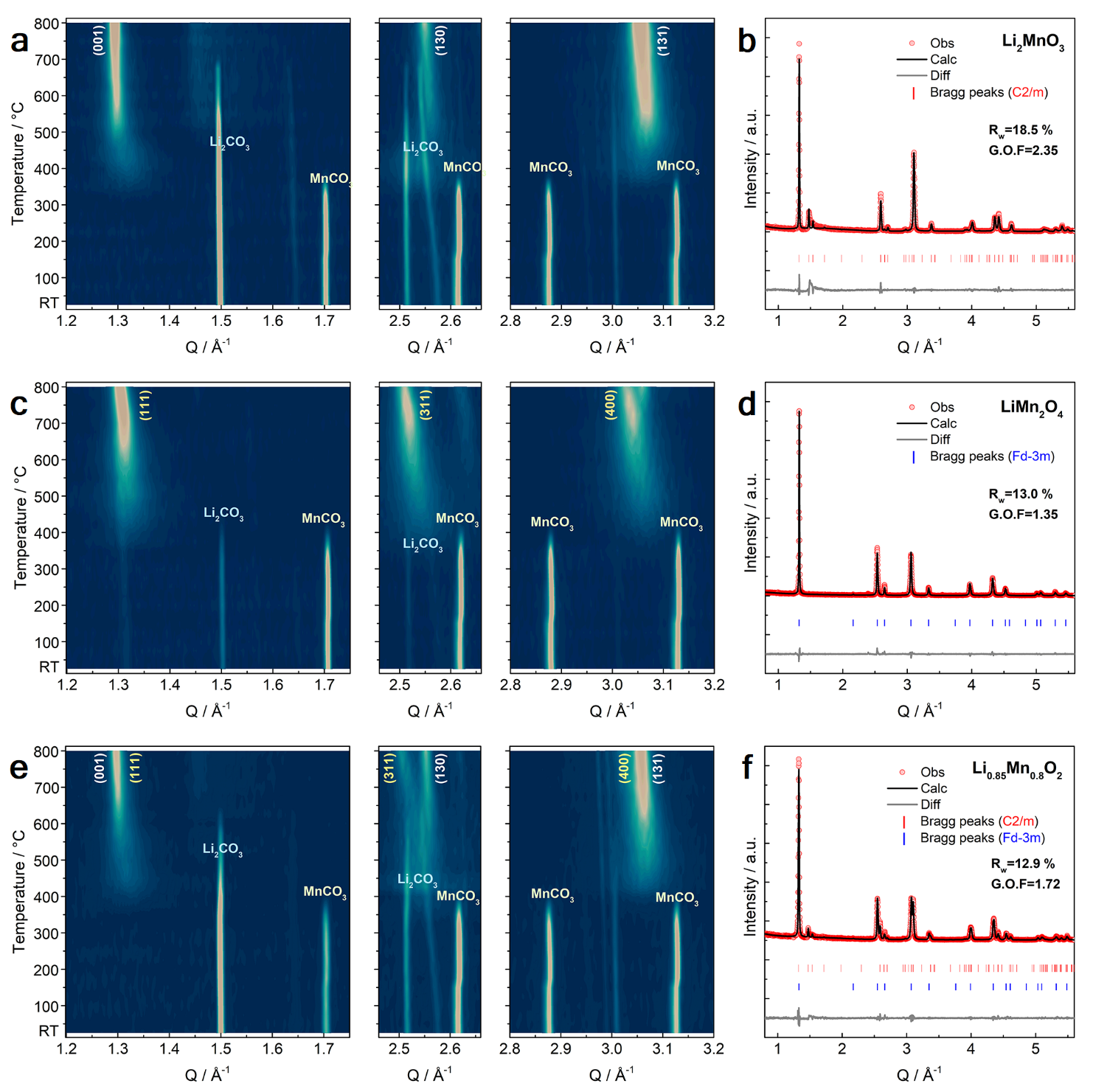


**Figure S4.** Structural evolutions during the syntheses of different lithium manganese oxides. (a) Contour maps of *in situ* VT-XRD patterns (λ=1.54 Å) during the synthesis of Li_2_MnO_3_. (b) XRD patterns (λ=1.54 Å) of Li_2_MnO_3_ prepared at 800 °C for 12h. (c) Contour maps of *in situ* VT-XRD patterns (λ=1.54 Å) during the synthesis of LiMn_2_O_4_. (d) XRD patterns (λ=1.54 Å) of LiMn_2_O_4_ prepared at 800 °C for 12h. (e) Contour maps of *in situ* VT-XRD patterns (λ=1.54 Å) during the synthesis of Li_0.85_Mn_0.8_O_2_. (b) XRD patterns (λ=1.54 Å) of Li_0.85_Mn_0.8_O_2_ prepared at 800 °C for 12h.

Table S4. Rietveld refinement parameters for O3-Li_2_MnO_3_

| **O3-Li_2_MnO_3_** Space group: C 2/m | | | | | | |
| --- | --- | --- | --- | --- | --- | --- |
| Lattice parameters:  a = 4.9287(3) Å, b = 8.5259(3) Å, c = 5.0224(8) Å; α = 90°, β = 109.11(1)°, γ = 90°; V_unit cell_ = 199.42(5) Å^3^; | | | | | | |
| Atom | Fractional coordinates | | | Multiplicity | Occupancy | U_iso_ |
|  | x | y | z |  |  |  |
| Li1 | 0 | 1/2 | 0 | 2 | 1 | 0.024(6) |
| Li2 | 0 | 0 | 1/2 | 2 | 1 | 0.020(5) |
| Li3 | 0 | 0.6606 | 1/2 | 4 | 1 | 0.020(5) |
| Mn | 0 | 0.1670(8) | 0 | 4 | 1 | 0.020(5) |
| O1 | 0.2189(1) | 0 | 0.2273(1) | 4 | 1 | 0.024(5) |
| O2 | 0.2540(1) | 0.3211(9) | 0.2233(1) | 8 | 1 | 0.024(5) |
| Refinement results: R_w_ = 18.5 %, G.O.F. = 2.35, reduced χ^2^ = 5.55 | | | | | |  |

Table S5. Rietveld refinement parameters for LiMn_2_O_4_

| **Spinel-LiMn_2_O_4_** Space group: F d-3m | | | | | | |
| --- | --- | --- | --- | --- | --- | --- |
| Lattice parameters:  a = b = c = 8.2210(1) Å; α = β = γ = 90°; V_unit cell_ = 555.61(4) Å^3^; | | | | | | |
| Atom | Fractional coordinates | | | Multiplicity | Occupancy | U_iso_ |
|  | x | y | z |  |  |  |
| Li | 1/8 | 1/8 | 1/8 | 8 | 1 | 0.044(3) |
| Mn | 1/2 | 1/2 | 1/2 | 16 | 1 | 0.033(2) |
| O | 0.2634(3) | 0.2634(3) | 0.2634(3) | 32 | 1 | 0.046(5) |
| Refinement results: R_w_ = 13.0 %, G.O.F. = 1.35, reduced χ^2^ = 1.81 | | | | | |  |

Table S6. Rietveld refinement parameters for Li_0.85_Mn_0.8_O_2_

| **Phase 1: Li_2_MnO_3_** | | | | Space group: C 2/m | | | at=36.4% | |
| --- | --- | --- | --- | --- | --- | --- | --- | --- |
| Lattice parameters:  a=4.9298(3) Å, b=8.5276(3) Å, c=5.0239 (5) Å; α=90°, β=109.06(7)°, γ=90°; V_unit cell_=199.61(9) Å^3^ | | | | | | | | |
| Atom | Fractional coordinates | | | | Multiplicity | Occupancy | | U_iso_ |
|  | x | y | z | |  |  |  |  |
| Li1 | 0 | 1/2 | 0 | | 2 | 1 | | 0.013(9) |
| Li2 | 0 | 0 | 1/2 | | 2 | 1 | | 0.013(9) |
| Li3 | 0 | 0.6606 | 1/2 | | 4 | 1 | | 0.013(9) |
| Mn | 0 | 0.1670(6) | 0 | | 4 | 1 | | 0.022(6) |
| O1 | 0.2134(2) | 0 | 0.2177(8) | | 4 | 1 | | 0.034(6) |
| O2 | 0.2500(5) | 0.3216(1) | 0.2134(5) | | 8 | 1 | | 0.034(6) |
| **Phase 2: Li_1.29_Mn_1.71_O_4_** | | | | Space group: F d-3m | | | at=63.6% | |
| Lattice parameters:  a = b = c = 8.1831(1) Å; α = β = γ = 90°; V_unit cell_ = 547.96(8) Å^3^; | | | | | | | | |
| Atom | Fractional coordinates | | | | Multiplicity | Occupancy | | U_iso_ |
|  | x | y | z | |  |  |  |  |
| Li1 | 1/8 | 1/8 | 1/8 | | 8 | 1 | | 0.013(9) |
| Li2 | 1/2 | 1/2 | 1/2 | | 16 | 0.145 | | 0.013(9) |
| Mn | 1/2 | 1/2 | 1/2 | | 16 | 0.855 | | 0.022(6) |
| O | 0.2584(1) | 0.2584(1) | 0.2584(1) | | 32 | 1 | | 0.034(6) |
| Refinement results: R_w_=12.9%, G.O.F.=1.72, Reduced χ^2^=2.96 | | | | | | | |  |


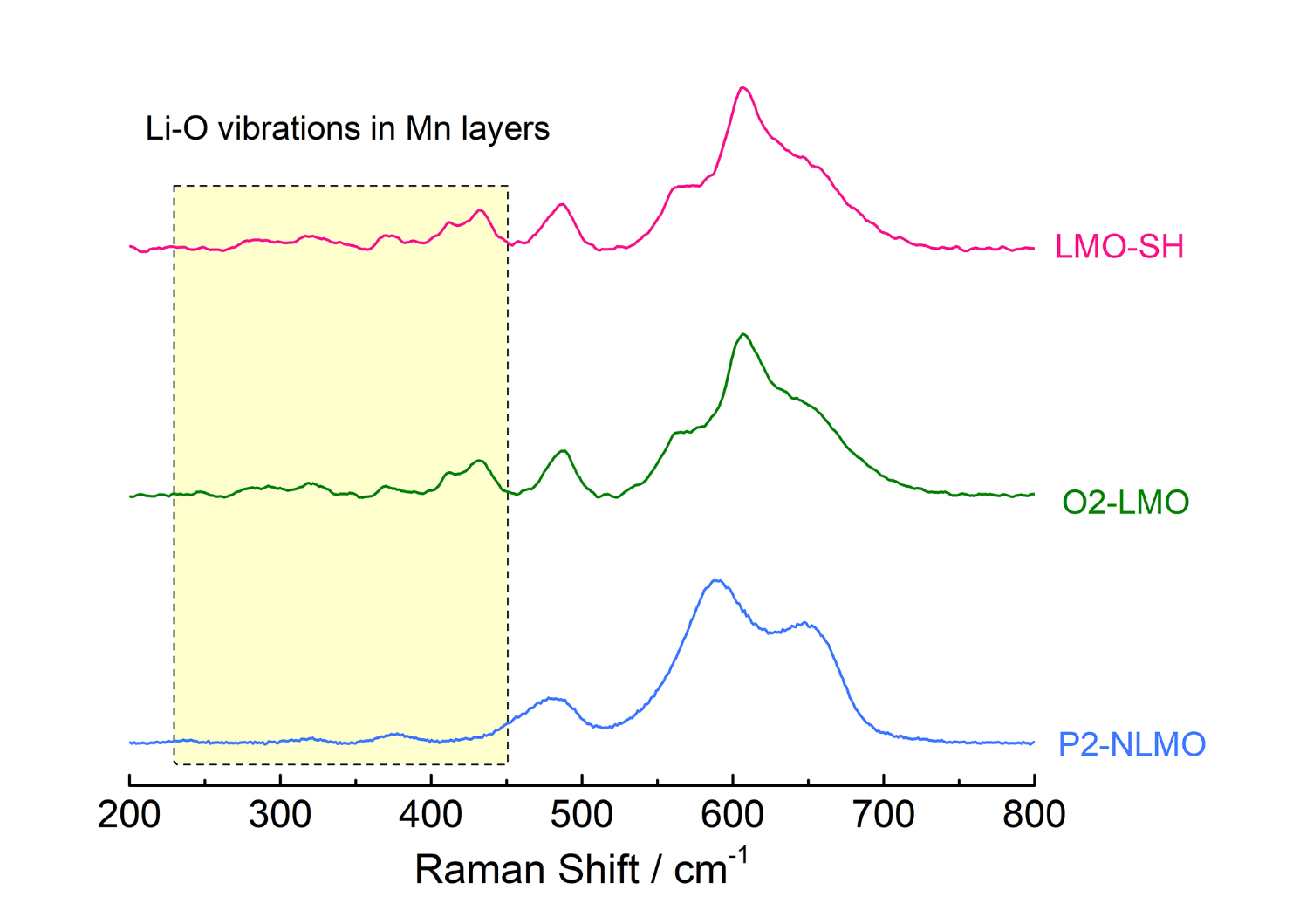


**Figure S5.** Raman spectra of P2-NLMO, O2-LMO and LMO-SH. Peaks in the region of 250-450 cm^-1^ indicate Li-O vibrations in Mn layers.


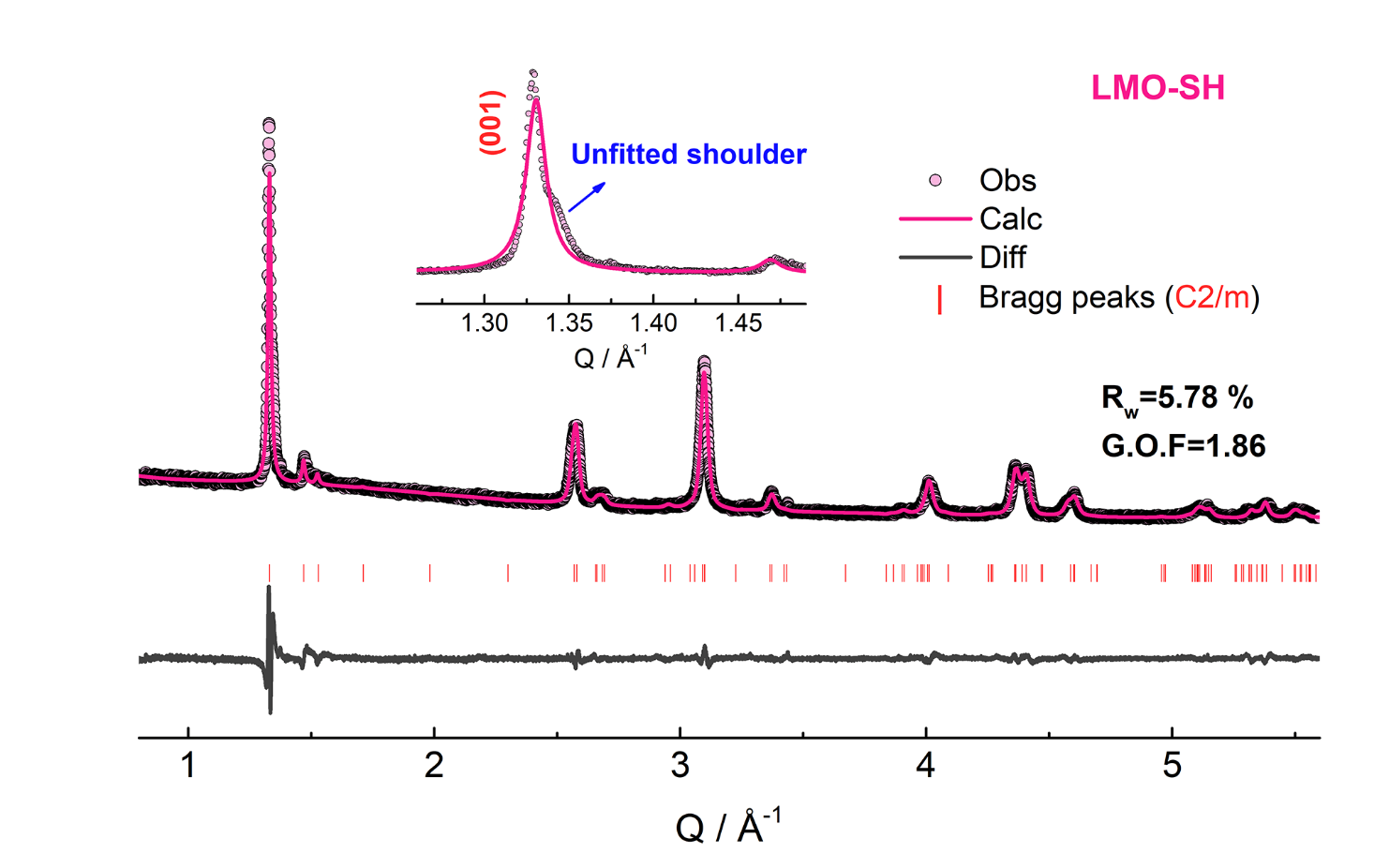


**Figure S6.** Synchrotron XRD patterns (λ=0.6887 Å) of LMO-SH. The Rietveld refinement was performed with a single monoclinic phase, and an unfitted shoulder can be observed over the right of the main (001) peak.

Table S7. Rietveld refinement parameters for LMO-SH with single monoclinic phase

| **O3-Li_0.852_Mn_0.792_O_2_** Space group: C 2/m | | | | | | |
| --- | --- | --- | --- | --- | --- | --- |
| Lattice parameters:  a = 4.9815(1) Å, b = 8.5544(2) Å, c = 5.0043(9) Å; α = 90°, β = 109.33(5)°, γ = 90°; V_unit cell_ = 201.22(8) Å^3^; | | | | | | |
| Atom | Fractional coordinates | | | Multiplicity | Occupancy | U_iso_ |
|  | x | y | z |  |  |  |
| Li1 | 0 | 1/2 | 0 | 2 | 0.375(4) | 0.029(6) |
| Li2 | 0 | 0 | 1/2 | 2 | 0.864(7) | 0.029(6) |
| Li3 | 0 | 0.6606 | 1/2 | 4 | 0.864(7) | 0.029(6) |
| Mn1 | 0 | 0.1685(7) | 0 | 4 | 1 | 0.032(5) |
| Mn2 | 0 | 1/2 | 0 | 2 | 0.564(6) | 0.032(5) |
| O1 | 0.1673(3) | 0 | 0.2054(2) | 4 | 1 | 0.032(2) |
| O2 | 0.1873(7) | 0.3260(4) | 0.2185(4) | 8 | 1 | 0.032(2) |
| Refinement results: R_w_ = 5.50 %, G.O.F. = 1.77, reduced χ^2^ = 3.13 | | | | | |  |

Table S8. Rietveld refinement parameters for LMO-SH with dual phases

| **Phase 1: Li_0.887_Mn_0.771_O_2_** | | | | Space group: C 2/m | | | at=95.2% | |
| --- | --- | --- | --- | --- | --- | --- | --- | --- |
| Lattice parameters:  a=4.9803(2) Å, b=8.5511(3) Å, c=5.0099(6) Å; α=90°, β=109.33(8)°, γ=90°; V_unit cell_=201.32(3) Å^3^ | | | | | | | | |
| Atom | Fractional coordinates | | | | Multiplicity | Occupancy | | U_iso_ |
|  | x | y | z | |  |  |  |  |
| Li1 | 0 | 1/2 | 0 | | 2 | 0.466(8) | | 0.033(4) |
| Li2 | 0 | 0 | 1/2 | | 2 | 0.881(4) | | 0.033(4) |
| Li3 | 0 | 0.6606 | 1/2 | | 4 | 0.881(4) | | 0.033(4) |
| Mn1 | 0 | 0.1696(9) | 0 | | 4 | 1 | | 0.033(8) |
| Mn2 | 0 | 1/2 | 0 | | 2 | 0.471(5) | | 0.033(8) |
| O1 | 0.2349(1) | 0 | 0.2274(8) | | 4 | 1 | | 0.026(1) |
| O2 | 0.1936(8) | 0.3354(9) | 0.2151(4) | | 8 | 1 | | 0.026(1) |
| **Phase 2: LiMn_2_O_4_** | | | | Space group: F d-3m | | | at=4.8% | |
| Lattice parameters:  a = b = c = 8.1063(6) Å; α = β = γ = 90°; V_unit cell_ = 532.69(3) Å^3^; | | | | | | | | |
| Atom | Fractional coordinates | | | | Multiplicity | Occupancy | | U_iso_ |
|  | x | y | z | |  |  |  |  |
| Li | 1/8 | 1/8 | 1/8 | | 8 | 1 | | 0.033(4) |
| Mn | 1/2 | 1/2 | 1/2 | | 16 | 1 | | 0.033(8) |
| O | 0.2529(7) | 0.2529(7) | 0.2529(7) | | 32 | 1 | | 0.026(1) |
| Refinement results: R_w_=3.81%, G.O.F.=1.23, Reduced χ^2^=1.50 | | | | | | | |  |


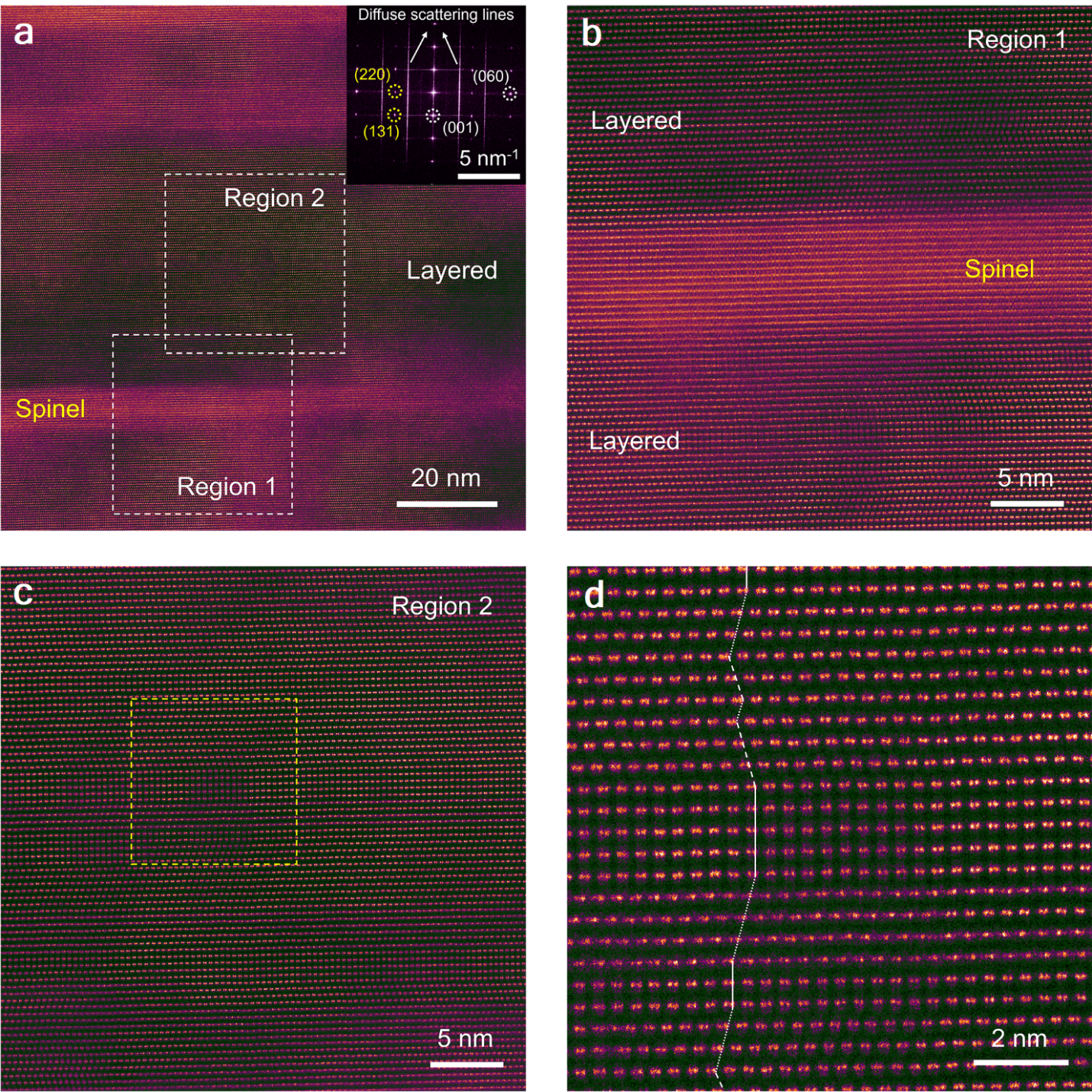


**Figure S7.** Structural characterization of LMO-SH. (a) HAADF-STEM images showing layered-spinel heterostructures in the bulk along the [100] zone axis. The inset shows corresponding FFT results, in which the diffuse scattering lines indicate the existence of stacking faults. Enlarged images of (b) region 1 and (c) region 2. (d) Enlarged image of the region marked by dashed yellow rectangular. The lines label different orientations along the *c*-axis, revealing multiple types of stacking faults.


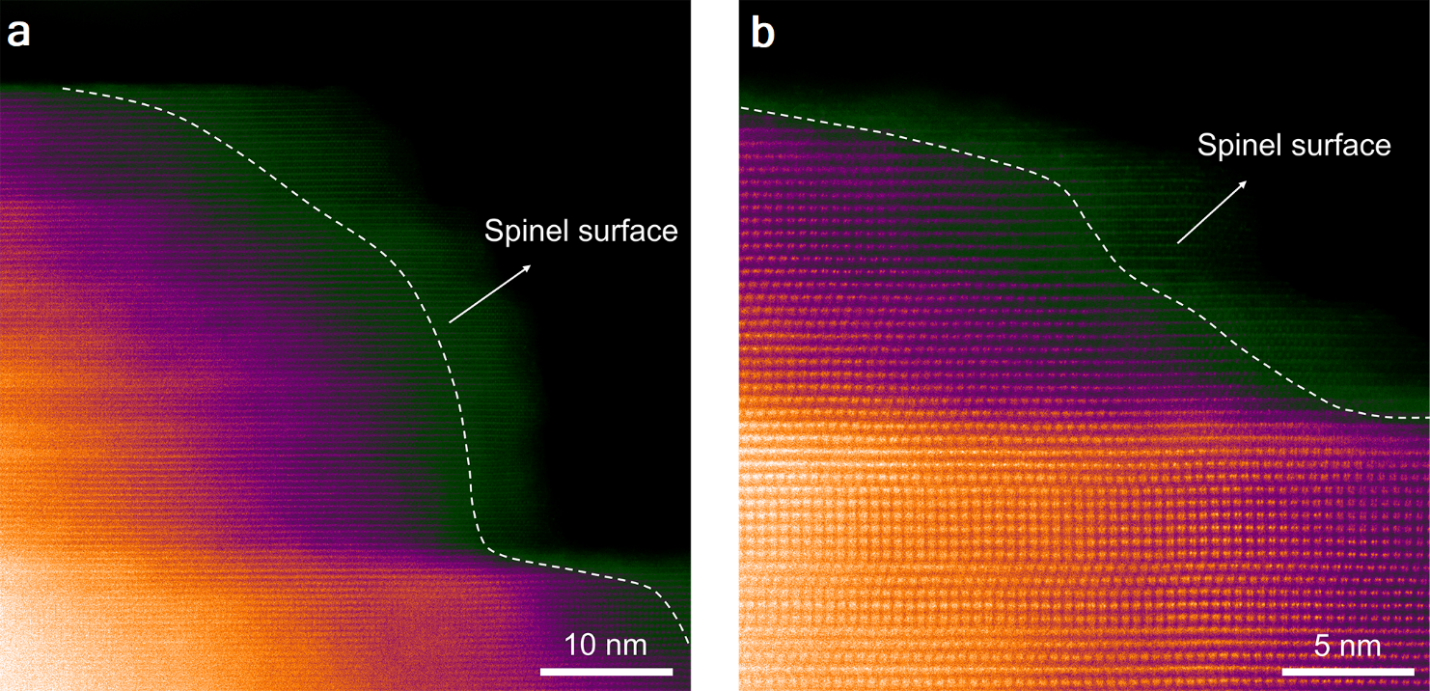


**Figure S8.** HAADF-STEM images of LMO-SH in the near-surface region. The dashed lines mark the boundaries between the layered phase in the bulk and the spinel surface.


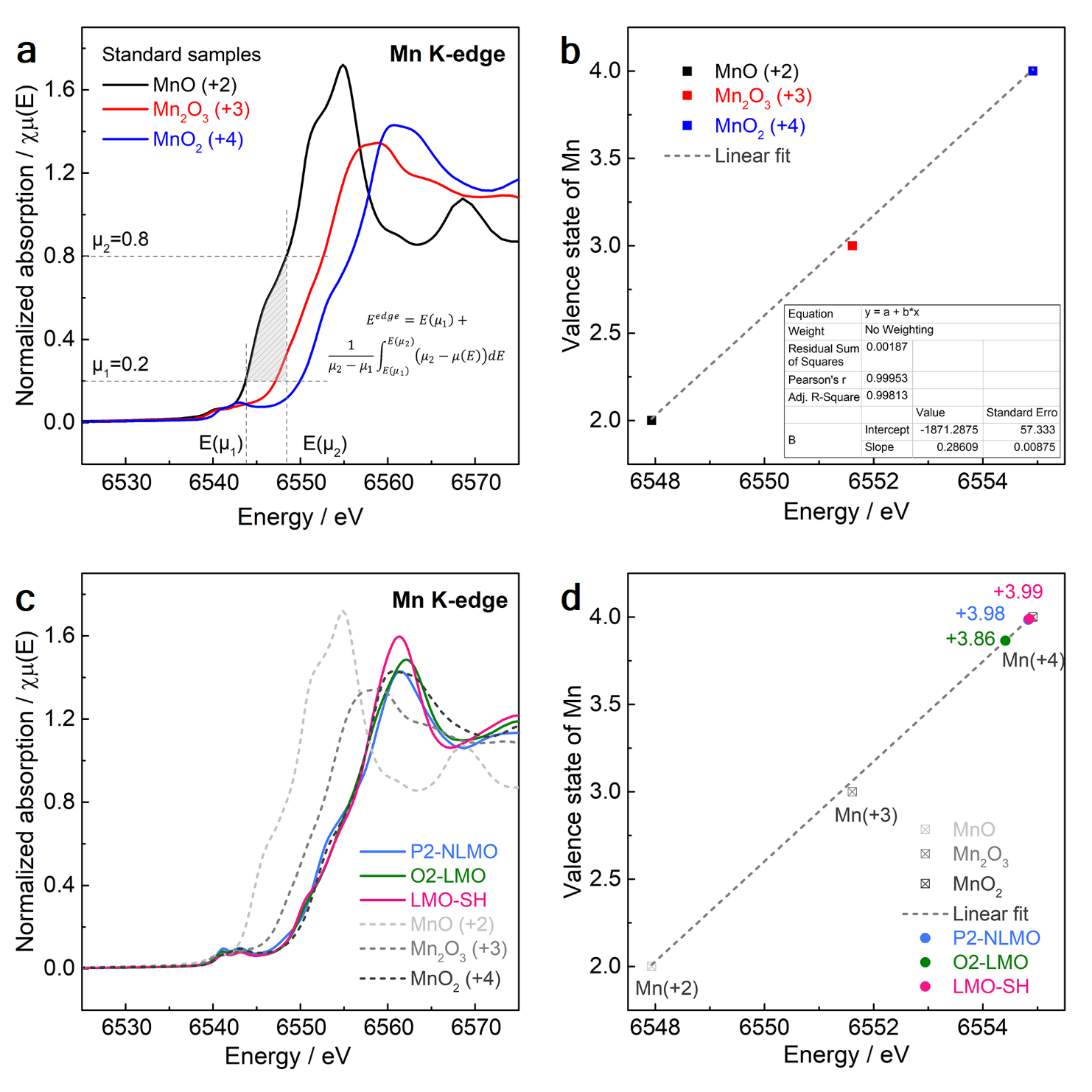


**Figure S9.** Mn K-edge XAS results. (a) Mn K-edge XANES results of standard samples. The grey region (μ=0.2-0.8) represents the area used for valence calculation. (b) Linear fit of K-edge energies for standard samples. (c) Mn K-edge XANES results of Mn for the as-prepared P2-NLMO, O2-LMO and LMO-SH powder samples, and (d) the correspondingly calculated valences of Mn.

Table S9. Calculated Mn K-edge positions and valence states of Mn

|  | **Sample name** | **^a^ Mn K-edge position** / eV | **^b^ Calculated valence of Mn** |
| --- | --- | --- | --- |
| Standard samples | MnO | 6547.93 | 2.01 |
|  | Mn_2_O_3_ | 6551.61 | 3.06 |
|  | MnO_2_ | 6554.92 | 4.00 |
| Powder samples | P2-NLMO | 6554.83 | 3.98 |
|  | O2-LMO | 6554.41 | 3.86 |
|  | LMO-SH | 6554.85 | 3.99 |
| LMO-SH electrodes  (1^st^ cycle) | OCV | 6554.30 | 3.83 |
|  | 1C-4.5 V | 6554.36 | 3.85 |
|  | 1C-4.8 C | 6553.41 | 3.58 |
|  | 1D-3.5 V | 6554.15 | 3.78 |
|  | 1D-3.0 V | 6553.87 | 3.71 |
|  | 1D-2.0 V | 6553.83 | 3.70 |
| LMO-SH electrodes  (2^nd^ cycle) | 2C-3.5 V | 6554.57 | 3.91 |
|  | 2C-4.5 V | 6555.19 | 4.08 |
|  | 2C-4.8 V | 6555.11 | 4.06 |
|  | 2D-3.5 V | 6554.27 | 3.82 |
|  | 2D-3.0 V | 6554.82 | 3.98 |
|  | 2D-2.0 V | 6554.48 | 3.88 |
| Notes: ^a^ The Mn K-edge positions were calculated according to the equation in **Figure S9a**. ^b^ The valence states of Mn were obtained via putting the Mn K-edge positions into the linear fit in **Figure S9b**. | | | |


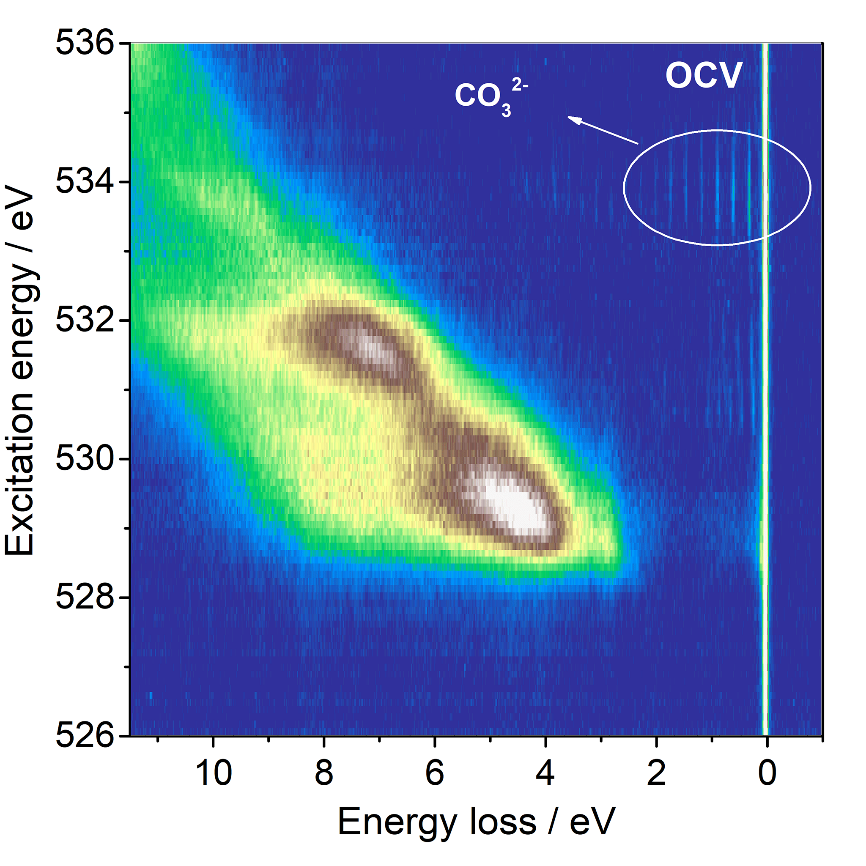


**Figure S10.** RIXS map of O K-edge for the LMO-SH electrode at OCV. Resonance peaks marked by the white circle represent typical resonance vibration signals from CO_3_^2-^, manifesting the existence of surface carbonate on the LMO-SH electrode.^9^


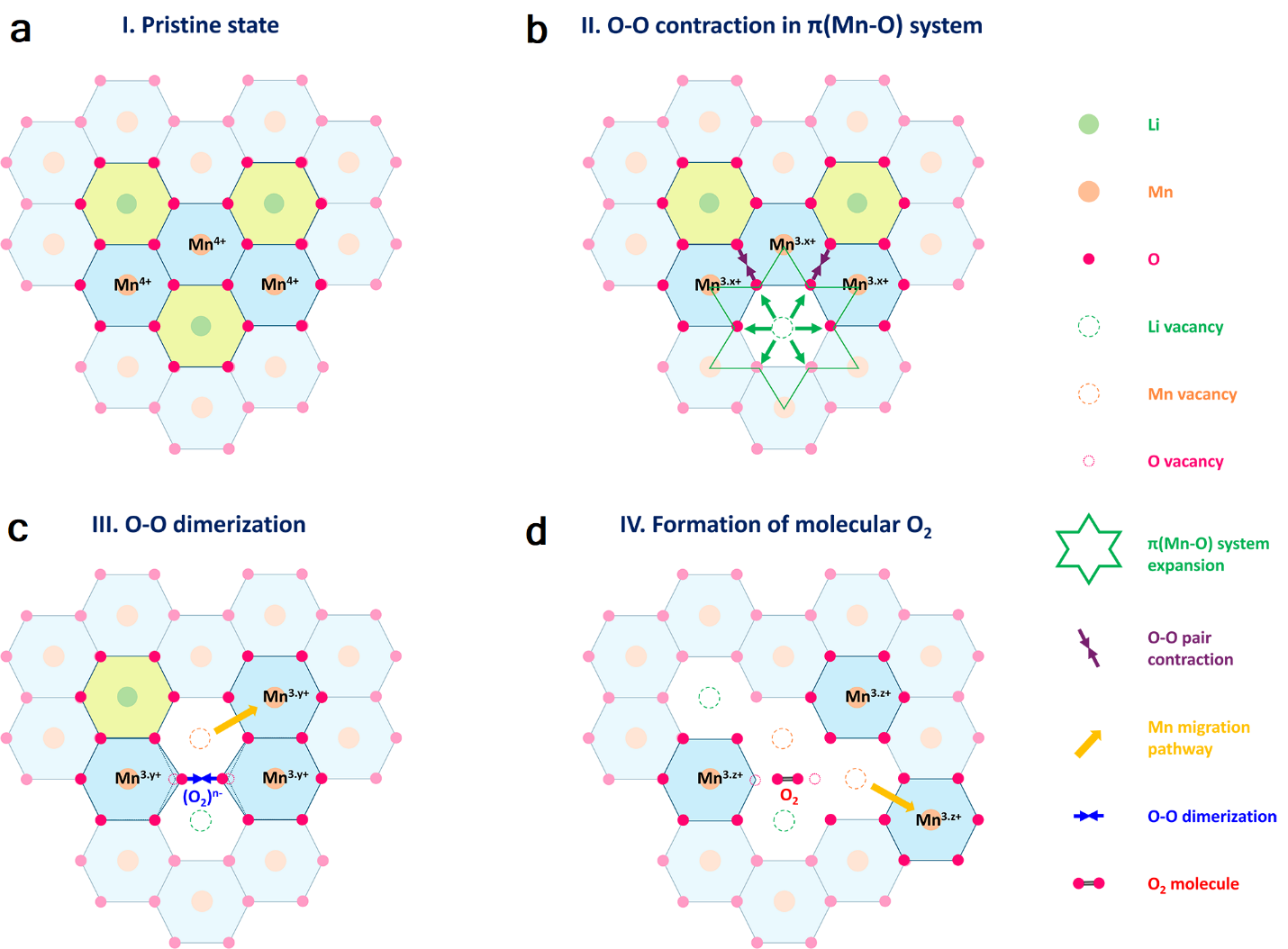


**Figure S11.** Schematic illustration of the reductive coupling mechanism upon oxygen redox.

In the pristine state, Mn is mainly in the oxidation state of 4+. At the beginning of the de-lithiation process, the redox center is the delocalized π(Mn-O) system, wherein the expansion of the Mn_6_ rings and the contraction of the O-O pairs takes place collectively. Such a process is supposed to be oxygen dominant, and the oxidation state of Mn decreases slightly to 3.x+. Upon the further removal of Li, Mn ions migrate to lithium vacancies, thereby promoting the O-O dimerization and even the formation of O_2_ molecules. In both processes, the oxidation state of the Mn decreases due to the de-coordination of the Mn-O bonds.


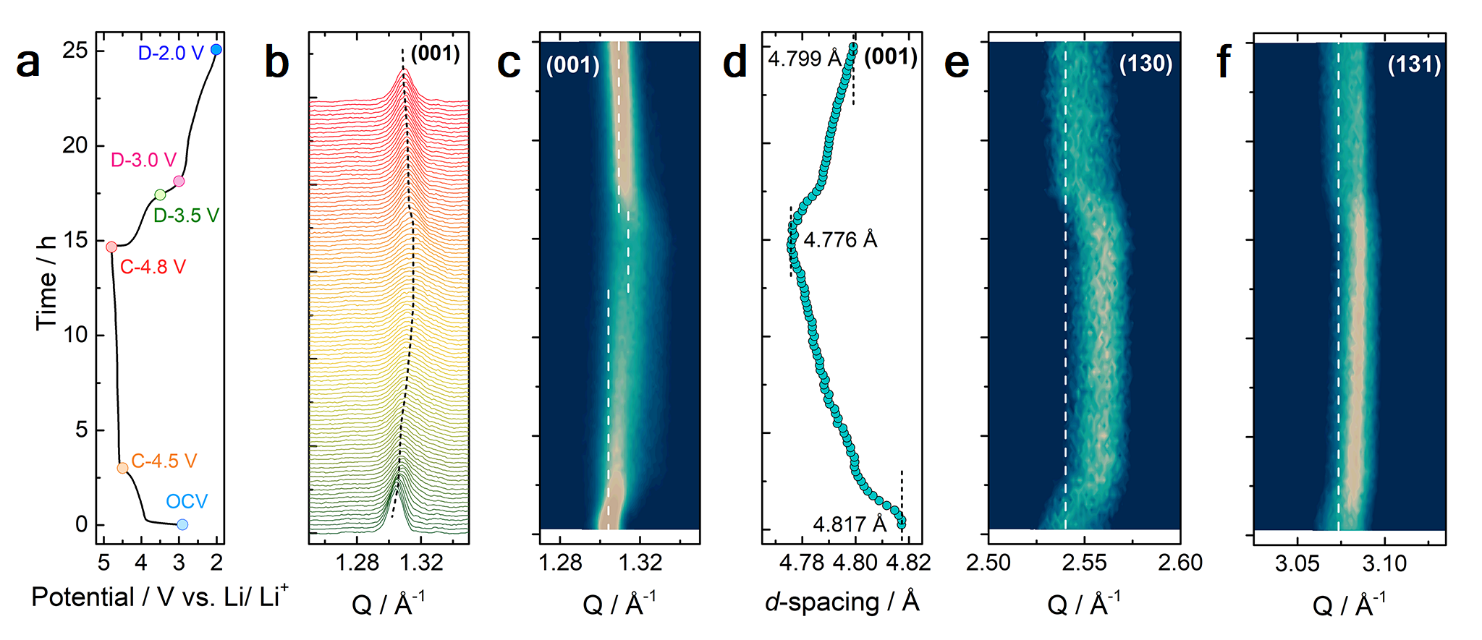


**Figure S12.** Structural evolution of LMO-SH in the 1^st^ cycle. (a) Voltage profile of the LMO-SH electrode. The current density is 10 mA/g, and the potential window is 2.0-4.8 V vs Li/Li^+^. (b-f) *In situ* XRD patterns (λ=1.54 Å) of the LMO-SH electrode. In (d), the calculated *d*-spacings reveal the structural changes along the *c*-axis, i.e., the lattice undergoes shrinkage and expansion in charge and discharge, respectively.


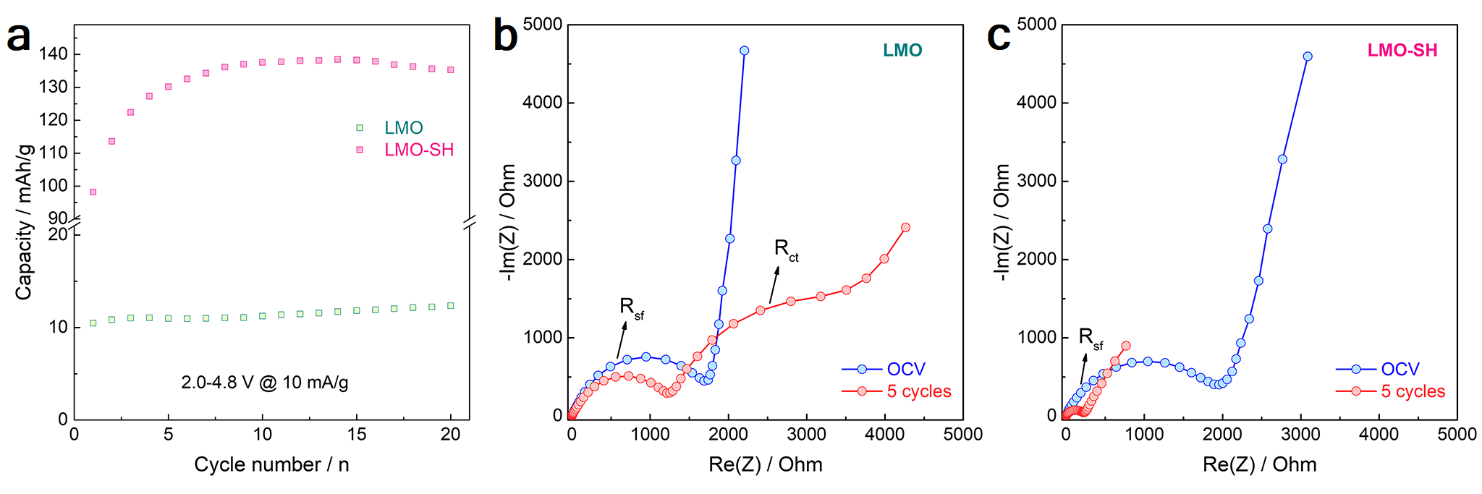


**Figure S13.** Electrochemical properties of LMO and LMO-SH. (a) Cycling performances of the LMO electrode and the LMO-SH electrode. The current density is 10 mA/g, and the potential window is 2.0-4.8 V vs. Li/Li^+^. Nyquist plots of (b) the LMO electrode and (c) the LMO-SH electrode at OCV and after 5 cycles (in the discharged state). R_sf_ and R_ct_ denote surface resistance and charge transfer resistance, respectively.

For both electrodes, the values of R_sf_ decrease after 5 cycles possibly due to the decomposition of surface carbonates. For the LMO-electrode, in the Nyquist plots, the emergence of a new semicircle in the middle frequency region represents the charge transfer resistance (R_ct_) in newly formed phases caused by surface degradations. It is not observed in the cycled LMO-SH electrode presumably because the spinel coating layers can suppress surface degradations.

**Supplementary References**

1. B. H. Toby, R. B. Von Dreele, *J. Appl. Cryst.* **2013**, *46*, 544-549.
2. J. P. Xu, Y. Xia, Z. Li, H. Chen, X. Wang, Z. Sun, W. Yin, *Nuclear Inst. and Methods in Physics Research* **2021**, *1013*, 165642.
3. K.-J. Zhou, A. Walters, M. Garcia-Fernandez, T. Rice, M. Hand, A. Nag, J. Li, S. Agrestini, P. Garland, H. Wang, S. Alcock, I. Nistea, B. Nutter, N. Rubies, G. Knap, M. Gaughran, F. Yuan, P. Chang, J. Emmins, G. Howell, *J. Synchrotron Radiat.* **2022**, *29*, 563-580.
4. G. Kresse, J. Furthmüller, *Phys. Rev. B* **1996**, *54*, 11169-11186.
5. J. P. Perdew, K. Burke, M. Ernzerhof, *Phy. Rev. Lett.* **1996**, *77*, 3865.
6. D. Eum, B. Kim, S. J. Kim, H. Park, J. Wu, S.-P. Cho, G. Yoon, M. H. Lee, S.-K. Jung, W. Yang, W. M. Seong, K. Ku, O. Tamwattana, S. K. Park, I. Hwang, K. Kang, *Nat. Mater.* **2020**, *19*, 419-427.
7. S. Grimme, J. Antony, S. Ehrlich, H. Krieg, *J. Chem. Phys.* **2010**, *132*, 154104.
8. G. A. Henkelman, B. P. Uberuaga, H. J. J. O. C. P. Jónsson, *J. Chem. Phys.* **2000**, *113*, 9901-9904.
9. C. Ma, J. Alvarado, J. Xu, R. J. Clément, M. Kodur, W. Tong, C. P. Grey, Y. S. Meng, *J. Am. Chem. Soc.* **2017**, *139*, 4835-4845.
